# Co-delivery of resolvin D1 and antibiotics with nanovesicles to lungs resolves inflammation and clears bacteria in mice

**DOI:** 10.1038/s42003-020-01410-5

**Published:** 2020-11-16

**Authors:** Jin Gao, Sihan Wang, Xinyue Dong, Leon G. Leanse, Tianhong Dai, Zhenjia Wang

**Affiliations:** 1 Department of Pharmaceutical Sciences, College of Pharmacy and Pharmaceutical Sciences, Washington State University, Spokane, WA 99202 USA; 2 Wellman Center for Photomedicine, Massachusetts General Hospital, Harvard Medical School, Boston, MA USA

**Keywords:** Drug delivery, Nanoparticles

## Abstract

Resolution is an active process that protects the host damage from inflammation responses induced by infections. Simultaneously resolving inflammation and eliminating pathogens may be effective to treat infectious diseases, but it is required to deliver therapeutics to infectious sites. Here, we proposed a strategy to incorporate RvD1 and an antibiotic (ceftazidime) in human neutrophil-membrane derived nanovesicles that can specifically target inflamed vasculature for treatment of lung infection caused by *P. aeruginosa*. Using the nitrogen cavitation method, we generated liposome-like nanovesicles from human neutrophil membrane. The results showed that nanovesicles loaded with RvD1 decreased cytokine levels and neutrophil lung infiltration, thus shortening the resolution intervals of lung inflammation. When RvD1 and ceftazidime were co-loaded in nanovesicles, they alleviated both inflammation and bacterial growth in the mouse lung. The studies reveal a new strategy to treat infectious diseases by designing nanoparticles to simultanesouly target host inflammatory pathways and pathogens.

## Abstract (web summary)

Gao et al. design a lung targeting nanovesicles made from human neutrophils membrane, and co-loaded inflammatory resolving mediator RvD1 and antibiotic CAZ inside the nanovesicles. These nanovesicles could accelerate the inflammation resolution and release the antibiotics to treat bacterial infections in mice. This approach is useful in developing multifunctional nanoparticle-based therapeutics for lung bacterial infections treatment.

## Introduction

*Pseudomonas aeruginosa* is a Gram-negative bacterium that causes infectious diseases including urinary and gastrointestinal tract infections, dermatitis, and bacteremia^1–3^. Lung infections by *P. aeruginosa* is very common in hospitals and is life threatening to patients with complicated conditions at a very high mortality^4^. Importantly, bacterial invasion to the lung can cause acute lung inflammation/injury (ALI)^5^. If inflammation responses are out of control, ALI may rapidly precipitate a severe form—acute respiratory distress syndrome (ARDS)—with mortality at 30–50%^6–9^. Usually, exhaustive antibiotics are administered to patients for life saving, but off-targeting and high doses of antibiotics cause systemic toxicity^10,11^. Most importantly, antimicrobial resistance quickly develops when the high dosage and frequency of antibiotics are administered^11,12^. Delivering antibiotics to infectious sites is highly demanding, to effectively treat infectious diseases, and it is possible to avoid the antimicrobial resistance^13,14^.

Infectious microenvironments include pathogens and inflammatory tissues^15,16^. For example, lung infection is a bacterial invasion via the airway. The lung has a unique structure, an alveolus that comprises blood capillaries attached to epithelia in the lung airspace. When bacteria invade in the airway, lung residence macrophages are activated to release cytokines (such as tumor necrosis factor-α (TNF-α) and interleukin-1β (IL-1β) that activate endothelium and neutrophils in the bloodstream. During the inflammation response, neutrophils adhere to activated endothelial cells to mediate neutrophil infiltration for bacterial clearance^6,7^. However, excessive/uncontrolled inflammation response may cause tissue injury, leading to a wide range of inflammatory disorders including ALI and sepsis^17–21^. Thus, targeting both pathogens and host inflammatory pathways existing in infectious microenvironments may be a novel approach to mitigate infectious diseases. We recently developed a method to generate cell membrane nanovesicles from HL60 cells using nitrogen cavitation. Intravital microscopy showed that the nanovesicles can specifically target inflamed endothelium and delivering anti-inflammatory drugs using nanovesicles alleviated vascular inflammation^22–24^.

Resolvins are lipid pro-resolving mediators enzymatically generated from docosahexaenoic acid (DHA), one type of endogenous ω-3 fatty acids, during the inflammation response, subsequently promoting the resolution of inflammation to protect tissue damage^25^. Resolvins activate neutrophil apoptosis and increase bacterial phagocytosis/efferocytosis, unlike other anti-inflammatory agents that only inhibit particular inflammatory factors^26,27^. RvD1 is a well-characterized lipid molecule, which blocks neutrophil infiltration and enhances cellular clearance of macrophages^28,29^. Endogenous generation of RvD1 is transient and its level is very low due to rapid degradation^30^. Delivering exogenous RvD1 may increase the inflammation resolution, but it is challenging to deliver RvD1 to inflammatory tissues in vivo.

Nanoparticle-based drug delivery systems include liposomes and polymer-based nanoparticles. To increase tissue selectivity and circulation times, nanoparticles are linked to targeting ligands and polyethylene glycol on nanoparticle surface. Advances in synthetic chemistry and bioengineering show the potential translation of synthetic nanoparticles, but their off-targeting and immunogenicity are still concerns due to the host clearance^31^. A new area is cell-based therapeutics via the utilization of cell membrane components for design of liposome-like nanovesicles and nanovesicles can evade the host clearance, such as red blood cell (RBC)-derived nanovesicles^32^. Loading polymeric nanoparticles^33^ or one type of drug^23^ to nanovesicles is the strategy to develop therapies.

Here we designed human neutrophil-membrane-derived nanovesicles loaded with both RvD1 and an antibiotic, which specifically targeted inflamed mouse lung endothelium to simultaneously activate inflammation resolution pathways and eliminate pathogens in treating lung bacterial infection. In vivo images showed that nanovesicles were accumulated in the inflamed lung after they were intravenously (i.v.) administered. Nanovesicles loaded with RvD1 decreased neutrophil lung infiltration, cytokine levels, and lung fluid permeability, and shortened the inflammation resolution intervals. When both RvD1 and ceftazidime (CAZ, an antibiotic) were delivered using nanovesicles to mice in the *P. aeruginosa*-induced lung infection model, the pathogen growth and inflammation responses were dramatically inhibited compared to several controls. Our studies provide a strategy to design bio-mimetic drug delivery platforms to synergistically target pathogens and inflammation pathways in infectious locations to treat infectious diseases.

## Results

### Design of neutrophil-derived nanovesicles to treat lung infections

**Fig. 1:**
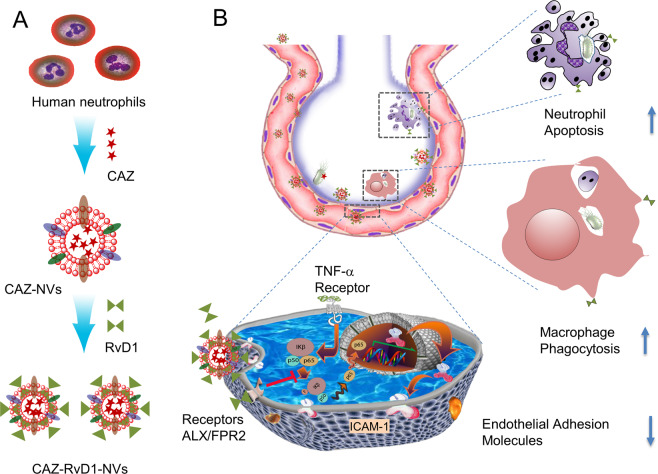
Schematic on development of human neutrophil membrane-derived nanovesicles to target the infectious lung. Scheme (**a**) shows the generation of neutrophil membrane-derived nanovesicles (NVs) co-loaded with RvD1 and Ceftazidime (CAZ). **b** The nanovesicles home to the infectious lung to deliver both RvD1 and CAZ. RvD1 bind to GPCRs (ALX/FPR2), inhibiting the NF-κB pathway to decrease the expression of cell adhesion molecules, and mitigating neutrophil tissue infiltration and increasing the phagocytosis. Simultaneously, CAZ inhibits bacterial growth in the lung.

Figure 1a is a schematic to illustrate the development on a drug delivery system made from human neutrophil cell membrane, which can co-load RvD1 in the membrane of nanovesicles and antibiotic (CAZ) inside the nanovesicles. Bacteria usually invade in the lung via the airway and subsequently activate macrophages to initiate the inflammation response^6,7^. During the inflammation response, lung endothelium is activated and highly express intercellular adhesion molecule-1 (ICAM-1) that binds to integrin β_2_ on neutrophils (most abundant white blood cells) in response to bacterial invasion^34^. Inspired by intercellular interactions between neutrophils and endothelium during lung infections, we have established an approach to generate liposome-like nanovesicles from human neutrophil membrane and nanovesicles may home inflamed lung endothelium, as nanovesicles possess cell adhesion proteins of their parent cells. RvD1 is a lipid molecule that can be incorporated in a lipid biolayer of nanovesicles and water-soluble antibiotics (such as CAZ) can be loaded inside the nanovesicles. The nanovesicles could deliver both RvD1 and CAZ to the infectious lung. Subsequently, RvD1 binds to G protein-coupled receptors (GPCRs), decreasing the expression of adhesion molecules on endothelium, promoting the neutrophil apoptosis and enhancing the phagocytosis. Simultaneously, CAZ could block bacterial proliferation in the lungs (Fig. 1b).

### Nanovesicles made from human neutrophils

We have recently developed a method of nitrogen cavitation to efficiently generate nanovesicles comprising cell plasma membrane and nanovesicles maintain tissue targeting of their parent cells^22,23^. Here we used primary human neutrophils to make nanovesicles using nitrogen cavitation (Supplementary Fig. S1). First, we collected the whole blood from healthy adults and neutrophils were isolated using a gradient density centrifugation method^35^. RBCs are as control after they are separated from leukocytes, as RBCs do not have the targeting feature to inflamed endothelium. We successfully obtained neutrophils at 5 × 10^5^ cells/ml with the purity higher than 95% (Supplementary Fig. S2A–C). Integrin β_2_ (Supplementary Figs. S2D and S3) on cell membrane was upregulated after neutrophils were treated with lipopolysaccharide (LPS)^36^.

**Fig. 2:**
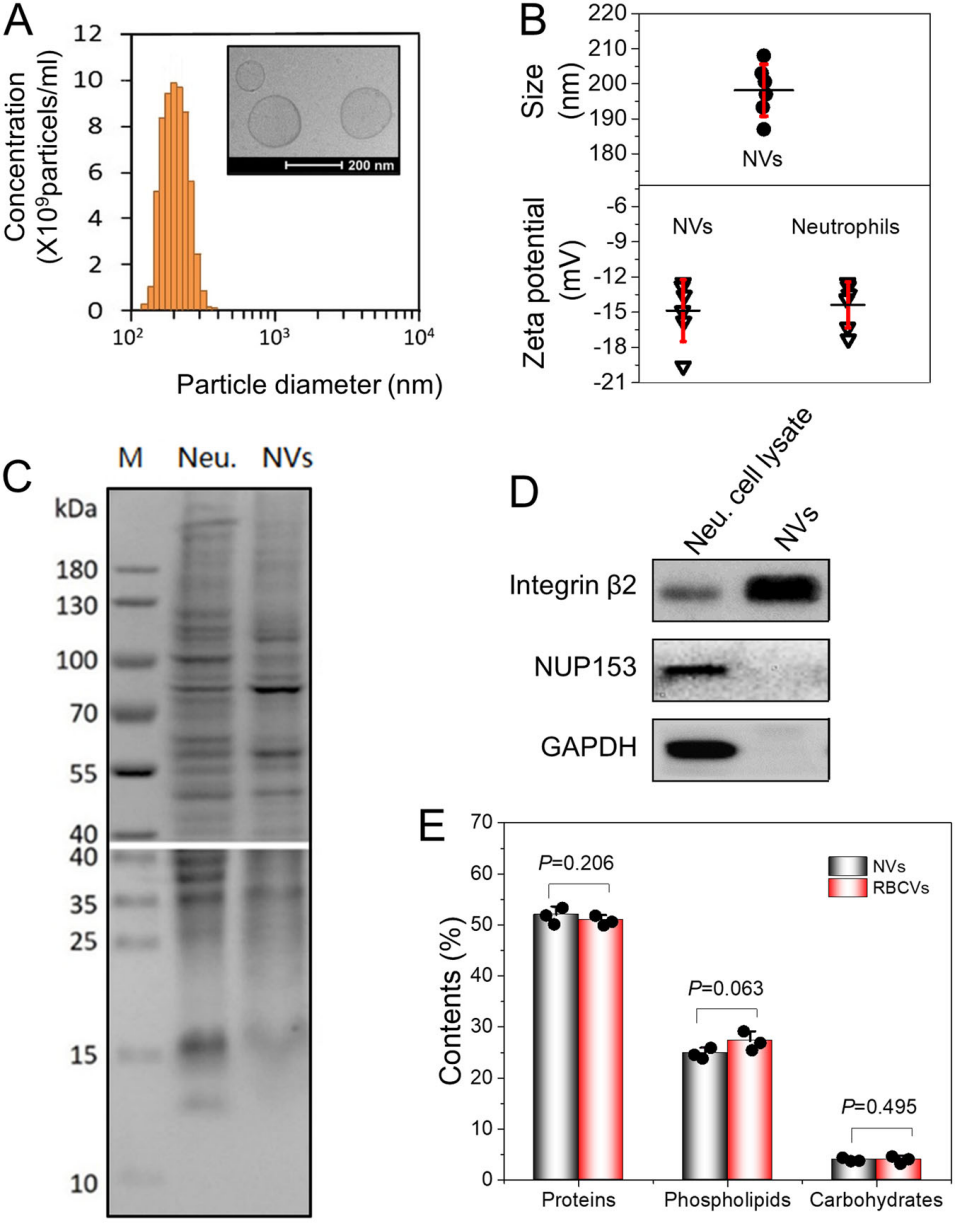
Characterization of NVs and RBCVs. **a** Size of NVs measured by DLS and cryo-TEM (inset). **b** The mean size and surface charges of NVs and their source cells measured by DLS. **c** Protein profiles of human neutrophils and NVs measured by SDS-PAGE at the same total protein loading of neutrophil lysis and NVs (*n* = 6 independent samples). **d** Western blot analysis for neutrophils and NVs. Fifty micrograms of each sample was loaded and western blottings were made using 15% SDS-PAGE. Neu denotes neutrophils. Integrin β_2_ (plasma membrane marker), GAPDH, and NUP153 (nuclear membrane marker) were detected. **e** Molecular compositions of NVs and RBCVs using BCA for proteins and colorimetry assays for phospholipids and carbohydrates, respectively (*n* = 3 independent samples). Data are presented as the mean ± SD.

Nanovesicles were formed when nitrogen cavitation force rapidly disrupted the cells (Supplementary Fig. S1). We made two types of nanovesicles, neutrophil-derived nanovesicles (called NVs) and RBCs-derived nanovesicles (called RBCVs). Dynamic light-scattering measurement showed that NVs had the size of 200 nm in diameter and cryogenic transmission electron microscopy (cryo-TEM) images indicated a liposome-like structure (Fig. 2a). Furthermore, the nanovesicle wall width was about 3–4 nm, suggesting that the nanovesicle layer was made of a lipid bilayer of cell membrane^37^. NVs had the zeta potentials of −14.9 ± 2.6 mv, similar to those of their parent cells (−14.4 ± 2.0 mv, *n* = 6) (Fig. 2b), implying that NVs had the similar surface property of their parent cells. We compared the protein profiles of NVs to those of their resource cells of neutrophils by SDS-polyacrylamide gel electrophoresis studies (Fig. 2c). The patterns of protein compositions in NVs followed their resource cells, but some proteins were higher or lower compared to those of the resource cells. This may be associated with the composition changes of NVs.

We also studied the major cell membrane protein of neutrophils, integrin β_2_^38^, and intracellular proteins, NUP153 (a nuclear membrane marker), and GAPDH using western blottings (Fig. 2d and Supplementary Fig. S4). The results indicated that NVs contained more integrin β_2_ compared to their parent cells of neutrophils. NUP153 was observed in neutrophils but not in NVs. Interestingly, GAPDH unlikely existed in NVs. The results indicated that the process of nitrogen cavitation can remove a nucleus and cytosol of a cell to form cell membrane vesicles, thus increasing the level of integrin β_2_ relative to that on neutrophils. In contrast, RBCs-derived RBCVs did not contain integrin β_2_ (Supplementary Figs. S5A–C and S6), thus RBCVs were a good control to assess the endothelial targeting of NVs to the inflamed lung. In addition, RBCVs had the size of 200 nm in diameter, and surface charges and protein profiles similar to their parent cells, RBCs (Supplementary Fig. S5A, B).

We also analyzed the molecular components of NVs and RBCVs (Fig. 2e). The results showed that NVs and RBCVs had similar compositions in proteins (51–52%), phospholipids (25–27%), and carbohydrates (4%). The results are consistent with cryo-TEM to show that nanovesicles were made of cell plasma membrane (Fig. 2a).

### NVs specifically target inflamed lungs

**Fig. 3:**
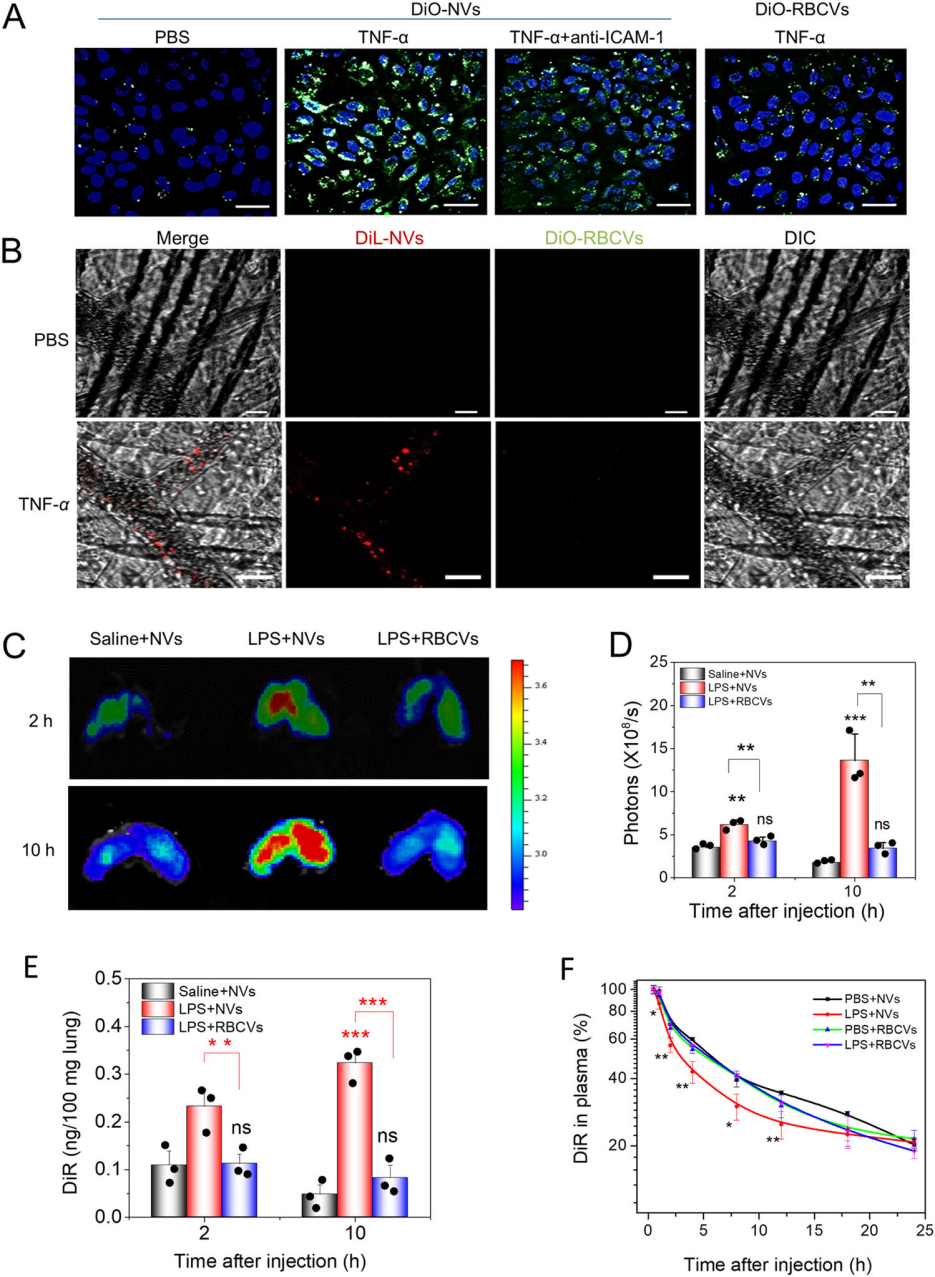
NVs target to inflamed vasculature in vitro and in vivo. **a** DiO-NVs or DiO-RBCVs (5 μg at protein) were incubated with activated or non-activated HUVECs (50 ng/ml TNF-α) under shaking condition (100 r.p.m.) and the images were taken using a fluorescence confocal microscope. Anti-ICAM-1 was used to block the binding of NVs to HUVECs. **b** Intravital microscopy visualized NVs and RBCVs in mouse cremaster vasculature. The images were taken simultaneously at 488 nm for DiO-labeling and at 560 nm for DiL-labeling, respectively. **c** IVIS images of mouse lungs after LPS (at 5 mg/kg) was intratracheally administered to the mice. **d** Quantitative analysis of fluorescent intensity of lung tissues based on the IVIS images as shown in **c**. **e** Nanovesicle fluorescence of lung homogenates was measured at 780 nm. **f** Pharmacokinetics of NVs and or RBCVs in healthy mice or inflamed mice induced by intratracheal LPS administration. Data are presented as the mean ± SD, *n* = 3 biologically independent experiments. *P < 0.05, **P < 0.01, ***P < 0.001 compared to control (saline or PBS) unless specified. The ns denotes nonsignificant difference.

First, we addressed whether NVs specifically bound to inflamed endothelium in vitro (Fig. 3a). The fluorescence confocal images showed that binding of NVs to endothelium increased after the endothelium was treated with 50 ng/ml TNF-α compared to that in the case of phosphate-buffered saline (PBS) treatment. When we blocked endothelial cells with 10 μg/ml anti-ICAM-1 antibodies, the signal of NVs was dramatically reduced. The result indicated that ICAM-1 was required for NVs to bind to inflamed endothelial cells. In contrast, RBCVs showed the moderate fluorescent signals compared to that of NVs when the endothelial cells were activated by TNF-α. Collectively, our studies showed that the binding of NVs to inflamed endothelium was required by the intercellular interactions between integrin β_2_ on NVs and ICAM-1 on endothelium.

To confirm whether this binding of NVs to inflammatory endothelium worked in vivo, we established a local inflammation on cremaster vasculature in a mouse model after intrascrotal injection of TNF-α. Three hours after the TNF-α injection, we i.v. administered both DiL-NVs and DiO-RBCVs, and imaged them in the post-capillaries of cremaster tissues. We observed that NVs bound to the vasculature, but RBCVs were not. Without the TNF-α challenge to cremaster tissues, we observed neither NVs nor RBCVs attached to the vasculature, implying that the vascular inflammation was required for the binding of NVs to the vasculature (Fig. 3b).

To determine whether NVs specifically targeted the inflamed lung, we intratracheally administered LPS (a bacterial toxin) to the lung to induce the local lung inflammation. We i.v. injected NVs and RBCVs, respectively, and investigated the lung targeting specificity of NVs using in vivo imaging system (IVIS). In healthy mice (without LPS challenge), it was unlikely that NVs accumulated in the lungs, but their accumulation was increased when the lungs were challenged with LPS (Fig. 3c). When we administered RBCVs to LPS-challenged mice, the accumulation of RBCVs was dramatically diminished. With increasing time, the fluorescence of NVs was increased in the mouse lungs. We quantitatively analyzed IVIS images (Fig. 3d), finding that the accumulation of EVs was increased by 5 folds compared with the  controls (RBCVs and without inflammation). This indicated that nanovesicles targeted to the mouse lungs during the lung inflammation. We also homogenized lung tissues and measured the fluorescence intensity of nanovesicles using a spectrometer. The results shown in Fig. 3e were consistent with the IVIS measurement. Collectively, NVs can specifically target inflamed lungs during lung infections.

The pharmacokinetics of nanovesicles were studied in the healthy and LPS-challenged mice (Fig. 3f). We observed that cell membrane-derived nanovesicles (NVs and RBCVs) had the long circulation in healthy mice and their dynamics were similar. However, the circulation of NVs became shorter when the lungs were challenged with LPS than that of RBCVs. This may be associated with the detour of NVs to the lungs because NVs interacted with inflamed lung vasculature as shown in Fig. 3c–e. Furthermore, we studied the biodistribution of NVs in healthy and LPS-induced lung inflammation mice (Supplementary Fig. S7). We still observed the majority of NVs in liver and spleen since nanoparticle delivery systems cannot overcome this challenge. However, we observed that LPS-induced lung inflammation increased the deposition of NVs in the lung compared to healthy mice, indicating that lung endothelial inflammation attracted NVs to diseased lung. This is consistent with the results in Fig. 3c–f.

In addition, the cytotoxicity studies of NVs in three human cell lines, such as human umbilical vein endothelial cells (HUVECs), normal human fibroblast (NHF), and human embryonic kidney 293T (HEK293T) cells, showed NVs did not cause cell death (Supplementary Fig. S8) even at the concentration of EVs at 250 μg/ml which was higher than the dose used in the following in vivo studies.

### NVs deliver RvD1 to enhance lung inflammation resolution

Leukocyte infiltration is a key process for immune surveillance to fight bacterial infections^34,39,40^, but persistent activation of neutrophils and their dysregulated tissue infiltration can cause organ malfunctions resulting in the death^21,41,42^. RvD1 is secreted in inflammatory lesions to self-resolve inflammation, but RvD1 quickly decreases^30^. Thus, delivering RvD1 in the inflammatory site may enhance inflammation resolution processes, such as limiting neutrophil infiltration and cytokine release during infections.

As NVs can specifically target inflamed endothelium in the infectious lungs, we addressed whether NVs were able to deliver RvD1 to enhance inflammation resolution. We loaded RvD1 in NVs via incubating RvD1 with NVs at 37 °C, as RvD1 possessed the lipid properties to allow the insertion of RvD1 to the lipid membrane of NVs (called RvD1-NVs). We found that we can achieve 2% (w/w) loading efficiency of RvD1 in NVs based on the measurement of high-performance liquid chromatography (HPLC)-mass spectroscopy (Supplementary Fig. S9). The size and surface charges of RvD1-NVs were similar to those of NVs, suggesting that RvD1 did not change the properties of NVs (Supplementary Fig. S10).

**Fig. 4:**
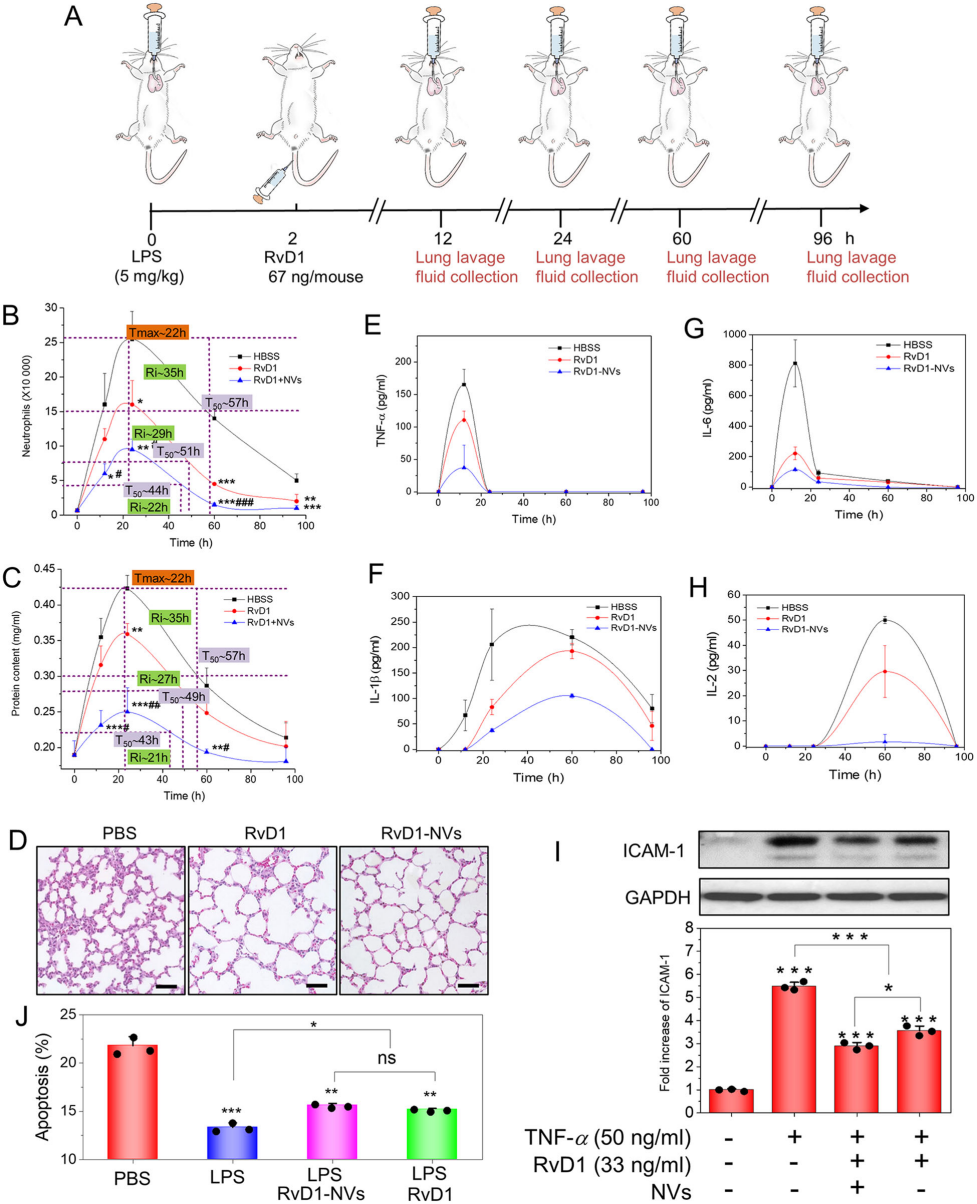
Enhanced resolution of lung inflammation by RvD1-NVs. **a** Schematic of an experimental protocol to evaluate the inflammation resolution in the acute lung inflammation (ALI) mouse model. Resolution indices of lung neutrophil infiltration (**b**) and protein concentrations in BALFs (**c**) in the ALI model. **d** Lung histology at 24 h after LPS treatment. Scale bar = 50 μm. **e**–**h** Cytokine profiles in BALFs in the ALI model. RvD1 at 67 ng/mouse was used in **b**–**h**. **i** Western blottings of ICAM-1 expression of HUVECs treated with or without TNF-α (50 ng/ml) or/and RvD1 (33 ng/ml) and RvD1-NVs, and their quantification. **j** Apoptosis of human neutrophils measured by flow cytometry after treatments with PBS, LPS (0.1 mg/ml), LPS (0.1 mg/ml)/RvD1 (33 ng/ml), and LPS (0.1 mg/ml)/RvD1-NVs (RvD1, 33 ng/ml). Data are presented as the mean ± SD, *n* = 3 biologically independent animals. *P < 0.05, **P < 0.01, ***P < 0.001 compared to control group at the same time point. ^#^*P* < 0.05, ^##^*P* < 0.01, ^###^*P* < 0.001 compared to free drug group at the same time point. The ns denotes nonsignificant difference. Copyright of the mouse images in **a** was obtained from Encapsula NanoSciences.

Figure 4a shows the experimental design to investigate the lung inflammation resolution by RvD1-NVs. In the experiments, RvD1-NVs were i.v. administered 2 h after the mice were challenged by LPS and then we collected lung bronchoalveolar lavage fluids (BALFs) to measure the dynamics of neutrophil (polymorphonuclear leukocytes (PMN)) infiltration, cytokines, and lung permeability. To quantitatively analyze the inflammation resolution, we used the resolution times defined in the literature^30^. *T*_max_ represents the time point when PMN numbers reach the maximum; *T*_50_ is the interval when PMN numbers reduce to 50% of the maximum; *R*_i_ (resolution interval) defined by *T*_50_ − *T*_max_ is the time period when 50% PMNs are lost. The resolution interval, *R*_i_ is a key factor to assess the inflammation resolution process. The shorter Ri represents the faster inflammation resolution. Figure 4b shows the time course of neutrophil infiltration. It was noted that Ri was decreased from 35 h in the case of Hankʼs balanced salt solution (HBSS) to 29 h after RvD1 administration. Interestingly, NVs-loaded with RvD1 shortened the *R*_i_ to 22 h and dramatically decreased neutrophil numbers in the lung compared to the cases of HBSS and free RvD1. The results indicated that NVs delivered RvD1 to inflamed lungs, blocking neutrophil infiltration and accelerating the inflammation resolution.

We also measured the total protein concentrations in the lungs, a parameter that represents lung vasculature injury and lung edema. The results in Fig. 4c showed the similar trend as that of neutrophil infiltration (Fig. 4b). RvD1-NVs shortened the Ri to 21 h compared to 27 h (free RvD1) and 35 h (HBSS). We observed that the protein accumulation in the lungs dramatically decreased after administration of RvD1-NVs, suggesting that RvD1-NVs inhibited the lung edema induced by LPS. The resolution times (*R*_i_) of neutrophil infiltration and lung edema are similar, suggesting that inhibition of neutrophil transmigration by RvD1-NVs may promote the repair of lung vascular injury induced by LPS. Furthermore, the lung histology (Fig. 4d) showed the lung vascular repair, consistent with the improved lung integrity after RvD1-NVs treatment as shown in Fig. 4c.

We also measured the time course of cytokines in the lung (Fig. 4e–h). RvD1-NVs significantly decreased cytokines (TNF-α, IL-6, IL-1β and IL-2) compared to the controls (HBSS and RvD1). Interestingly, the patterns of each cytokine were different; TNF-*α* and IL-6 occurred in the early time, but IL-1β and IL-2 reached the maximums in the late time.

Furthermore, we studied whether RvD1 regulated the ICAM-1 expression of endothelial cells to inhibit neutrophil lung infiltration. Fig. 4i (Supplementary Fig. S11) shows that RvD1 can downregulate ICAM-1 in the pre-TNF-α-treated endothelial cells after they were treated with RvD1 compared to the control. When RvD1-NVs were used, the ICAM-1 expression was further decreased. The results suggested that RvD1-NVs can target lung endothelium to inhibit the ICAM-1 expression via nuclear factor-κB (NF-κB) pathway and this inhibition may prevent neutrophil infiltration, thus contributing to the inflammation resolution. We also studied the apoptosis of neutrophils in vitro after they were treated with RvD1 (Fig. 4j). LPS treatment decreased the neutrophil death, but RvD1 or RvD1-NVs accelerated their death via apoptosis^28^, thus resolving inflammation responses. This is consistent with the reduction of neutrophil lung infiltration after RvD1 treatment (Fig. 4b).

### Enhanced delivery of antibiotics to the lung using NVs

**Fig. 5:**
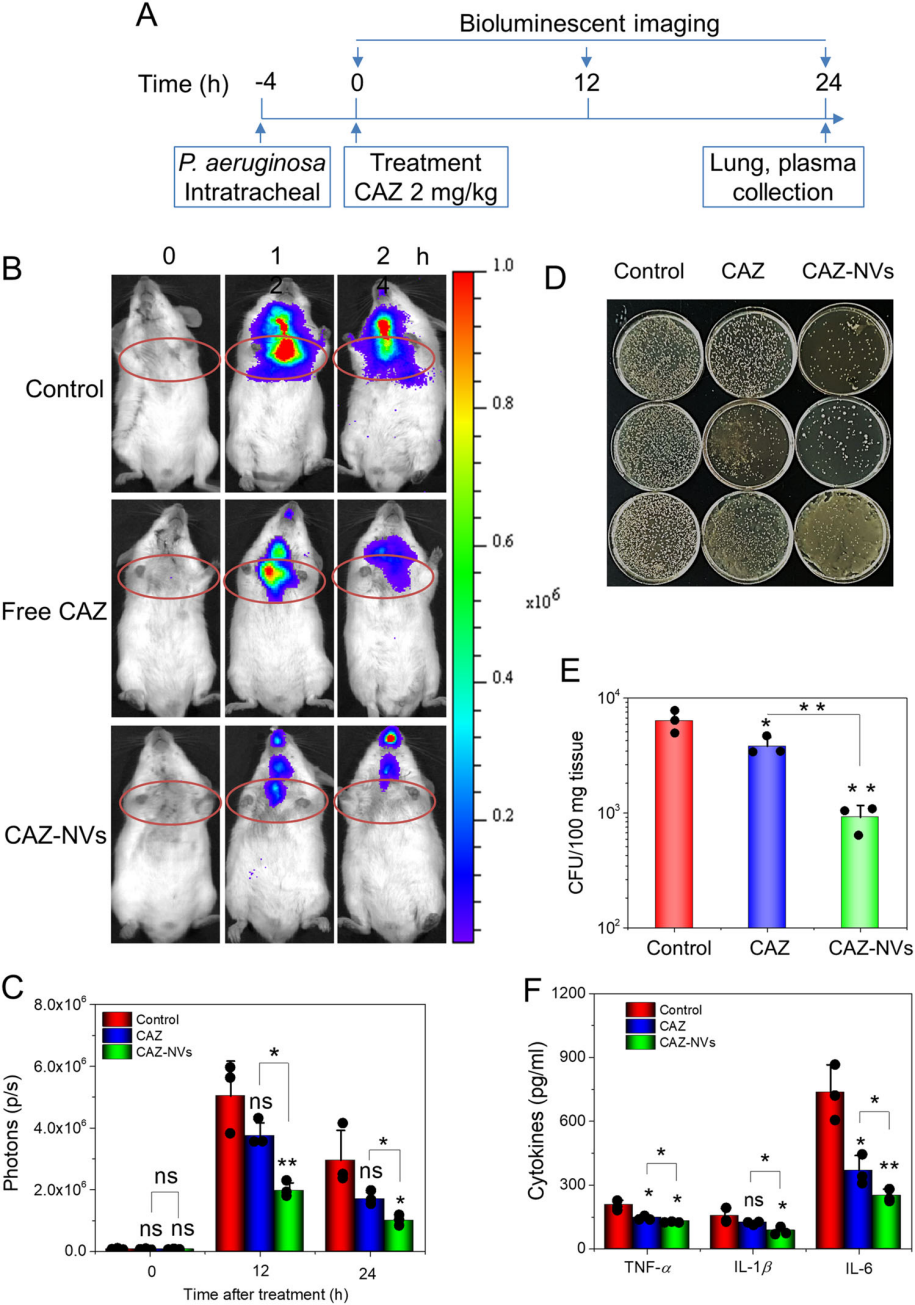
CAZ-NVs inhibit the growth of *P. aeruginosa* PAO1-Luc in the mouse lung. **a** The animal experiment protocol in *P. aeruginosa* PAO1-Luc lung infection. The animals were intratracheally distilled 5 × 10^5^ CFU bioluminescent *P. aeruginosa* PAO1-Luc followed with the treatment with CAZ or CAZ-NVs at 2 mg/kg of CAZ. The bioluminescent images were taken by IVIS. **b** Representative bioluminescent images of *P. aeruginosa* PAO1-Luc in the lungs. **c** Quantitative analysis of the bioluminescent IVIS images of **b**. CFUs in homogenates of lungs (**d**) and their quantifications at 24 h after the CAZ treatment (**e**). In **d**, the row represents three times of experiments. **f** Cytokines, TNF-α, IL-1β, and IL-6, in BALFs at 24 h after CAZ treatment. Data are presented as the mean ± SD, *n* = 3 biologically independent animals. *P < 0.05, **P < 0.01, ***P < 0.001 compared to control (PBS) unless specified on the figure. The ns denotes nonsignificant difference.

Bacterial infections to the lung cause the vascular inflammation response by upregulation of ICAM-1 on endothelium^34^, thus NVs may increase the delivery of antibiotics to the infection site. To image bacterial infections in the lung, we intratracheally administered the bioluminescent *P. aeruginosa* (Supplementary Fig. S12) to the mouse lung, and then we imaged bacterial growth in live mice using IVIS. CAZ is a water-soluble agent, and it can be loaded inside nanovesicles in the process of making cell membrane nanovesicles using nitrogen cavitation. The CAZ was quantified by HPLC (Supplementary Fig. S13) and the release of CAZ was studied at 4 °C–37 °C or in the fetal bovine serum (FBS) condition (Supplementary Fig. S14). The results showed that CAZ can be loaded inside NVs and the drug release was sustained. We i.v. administered CAZ-NVs to the mice after *P. aeruginosa* was locally administered to the lung. The experimental design was shown in Fig. 5a. The real-time lung bacterial infection was imaged by IVIS as shown in Fig. 5b. We observed that *P. aeruginosa* growth in the lung, but CAZ-NVs inhibited the bacterial growth. The quantitative analysis of images (Fig. 5c) showed that CAZ-NVs were more potent to prevent bacterial growth compared to the controls (HBSS and free CAZ). Furthermore, we collected lung lavage fluids and cultured bacteria to count the colonies (Fig. 5d, e), showing that the bacterial growth was dramatically inhibited by CAZ-NVs, consistent with the IVIS imaging results (Fig. 5b, c). In addition, we measured the cytokines in the lung lavage and the results (Fig. 5f) showed that CAZ-NVs also mitigated cytokine production because of decreased bacterial growth.

### Co-delivery of RvD1 and CAZ resolves inflammation and eliminates bacteria in the lung

We have demonstrated that NVs delivered either RvD1 to promote the inflammation resolution pathways or delivered CAZ to stop the bacterial growth in the mouse lung. Bacterial infections include two components: pathogens (bacteria) and the host inflammation response present in infection microenvironments. Therefore, delivering both RvD1 and CAZ to infectious lesions may be a new approach to treat bacterial infections.

**Fig. 6:**
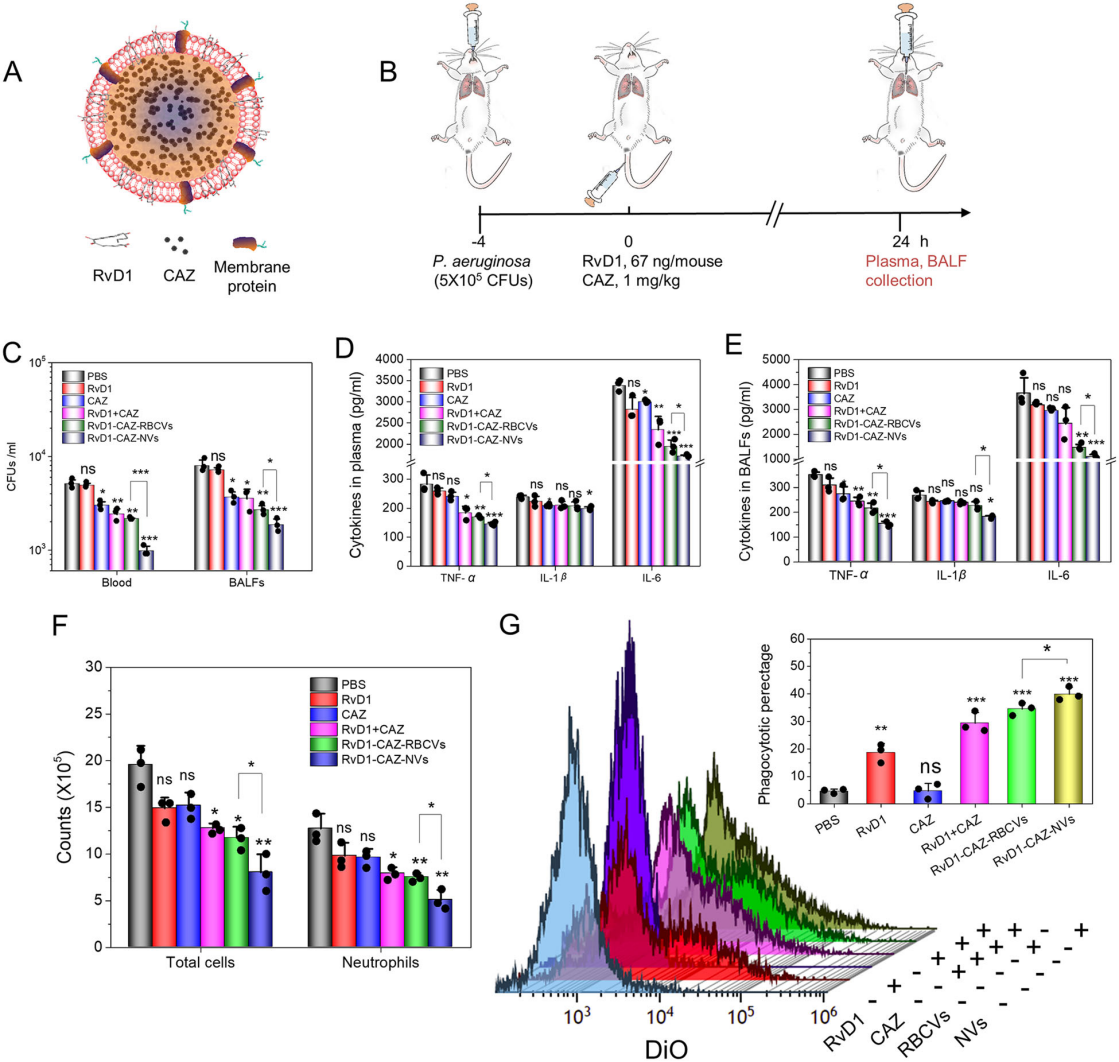
RvD1-CAZ-NVs increase the delivery of RvD1 and CAZ in the infectious lung. **a** RvD1-CAZ-NVs comprised CAZ inside nanovesicles and RvD1 in the lipid layer of nanovesicles. **b** Scheme of the animal experimental protocol. CFUs of bacteria in blood and BALFs (**c**), and cytokines (TNF-α, IL-1β, and IL-6) in the blood (**d**) and BALFs (**e**). **f** Total cells and neutrophils in BALFs. **g** Bacterial phagocytosis by macrophages was quantified by flow cytometry and their quantitative analysis. Data are presented as the mean ± SD, *n* = 3 biologically independent experiments. *P < 0.05, **P < 0.01, ***P < 0.001 compared to control (PBS) unless specified on the figure. The ns denotes nonsignificant difference. Copyright of the mouse images in **b** was obtained from Encapsula NanoSciences.

To prove this idea, we co-loaded CAZ inside nanovesicles and RvD1 entrapped in the lipid membrane of nanovesicles (as shown in Fig. 6a). We observed that loading of CAZ and RvD1 did not change the size and surface charges of nanovesicles (Supplementary Fig. S15). The experimental design was illustrated in Fig. 6b. After we administered *P. aeruginosa* in the lung airway, and the lung inflammation responses activate endothelium to express ICAM-1. Thus, NVs can target infected lungs to deliver both RvD1 and CAZ. We measured bacterial numbers in blood and BALF after the mice were treated with varied formulations (Fig. 6c), and the results showed that RvD1-CAZ-NVs were more potent than all other controls to inhibit the bacterial growth. Similarly, we quantitatively analyzed cytokines in blood and BALF (Fig. 6d, e), and neutrophil lung infiltration (Fig. 6f). Collectively, the results demonstrated that RvD1-CAZ-NVs significantly controlled bacterial infections compared to RvD1-CAZ-loaded RBCVs and combined RvD1 and CAZ. This indicated that NVs can deliver both RvD1 and CAZ to infectious vasculature, thus mitigating inflammation responses and bacterial proliferation.

RvD1-CAZ-NVs showed the dramatic reduction of bacterial loading in blood and BALF (Fig. 6c), suggesting that either RvD1 or CAZ was increased in inflammatory tissues responsible for bacterial reduction. The studies^30^ show that RvD1 can increase the phagocytosis of macrophages to contain bacteria. We performed in vitro experiments as shown in Fig. 6g, showing that RvD1 promoted the phagocytosis to destroy bacteria. The result suggests that NVs may increase the delivery of RvD1 in the inflammatory lungs that may place some roles in bacterial clearance in the lung infection.

## Discussion

Acute inflammation is a self-limited protective response against invading microbes or tissue injury and is tightly controlled by the host via actively generating specialized pro-resolving mediators. For example, RvD1 can help the inflammatory tissues return to homeostasis through the inflammation resolution process^26^. RvD1 is a critical member of pro-solving agents and is biosynthesized from DHA, one type of Omega-3 fatty acids. The studies showed that RvD1 specifically bind to immune cells and activates two separate GPCRs, named ALX and GPR32. RvD1 enhanced the phagocytic and clearance activities of human monocytes via the GPCRs^26,29,43,44^. In addition, through directly binding to the receptors, RvD1 displayed potent and stereoselective anti-inflammatory actions, such as limiting neutrophil infiltration and cytokine secretion. Furthermore, RvD1 actions to human PMNs can decrease actin polymerization and block LTB4-regulated adhesion molecules (including integrin β_2_)^45^. Therefore, RvD1 is a potent regulator to control immune response to the inflammation. However, biosynthesis of RvD1 in inflammatory tissues is transient and rapidly disappears due to its degradation^30^. Delivering RvD1 to inflammatory tissues is needed to maintain the therapeutic dosage in persistently activating inflammation resolution circuits.

Lung infections are severe inflammatory diseases. The pathogenesis is involved with invading pathogens and the host inflammation response. *P. aeruginosa* is a major bacterium to infect the lung. When bacteria accumulate in the lung, the lung resident macrophages initiate the inflammation response including cytokine secretion, endothelial activation, and neutrophil transmigration to the lung^34^. The dysregulated inflammation response can cause ALI. If the inflammation response is persistent, ALI may quickly become its severe form, ARDS, with the mortality at 30–50%^6,7^. Administration of antibiotic is a major tool to treat bacterial infections, but cannot target the host inflammatory pathways. Moreover, current approaches to deliver antibiotics cannot specifically target infectious tissues, thus possibly leading to the systemic toxicity and increased antimicrobial resistance^10,12^. It is needed to develop innovative approaches to simultaneously target pathogens and inflammatory pathways inside infectious tissues.

We have designed cell membrane-derived nanovesicles that were loaded with RvD1 and CAZ that can target inflammatory pathways and bacteria, respectively. During lung infections, lung endothelial cells activate to express ICAM-1 that can bind to integrin β_2_ on activated neutrophils. Inspired with the unique intercellular interactions, we employed nitrogen cavitation to generate plasma membrane-formed nanovesicles from human neutrophils and this structure was proved by the cryo-TEM images and molecular compositions of neutrophil nanovesicles (Fig. 2). Compared to the nanovesicles made from RBCs, we found that neutrophil nanovesicles (NVs) can specifically target to inflamed lung endothelial cells during lung inflammation induced by LPS (Fig. 3).

To study the molecular mechanism in which RvD1 controls the inflammation, we loaded RvD1 in the lipid layer of NVs and studied the time course of inflammation responses, such as neutrophil infiltration and cytokine secretion. We observed that delivering RvD1 using NVs enhanced the speed of inflammation resolution by 1.6 times compared to without treatment (Fig. 4b). In addition, neutrophil infiltration and cytokine levels were dramatically decreased (Fig. 4e–h). Most importantly, we observed that RvD1-NVs can also accelerate lung repair from edema by 1.7 times compared to without treatment (Fig. 4c). The lung histology (Fig. 4d) strongly support the conclusion that RvD1-NVs target inflammatory lungs to enhance the resolution of lung inflammation. Furthermore, the RvD1 can target the NF-κB pathway in endothelial cells to inhibit ICAM-1 expression (Fig. 4i and Supplementary Fig. S11). Collectively, RvD1 regulates the inflammation response of neutrophils and endothelial cells to activate the resolution circuits of inflammation.

When co-delivery of RvD1 and CAZ using NVs to the mouse lungs in the bacterial infection model, we found that RvD1-CAZ-NVs dramatically decreased bacterial proliferation and inflammatory response (Fig. 6c–f). The reduction of bacteria may be associated with the enhanced phagocytosis of neutrophils and macrophages (Fig. 6g). This is consistent with the observation that RvD1 can lower antibiotics to treatment bacterial infection^30^.

Human NVs used in the mouse studies may have some concerns on potential immunogenicity. However, our goal is to examine whether human neutrophil nanovesicles can target inflamed vasculature during inflammation or bacterial infections. In addition, we did not observe the side effect on mouse models (such as mouse behavior and weight changes) after we administered NVs or RBCVs. Our in vitro toxicity studies on several cells (Supplementary Fig. S8) did not show the cell death. The current data suggest that human cell-derived nanovesicles do not show observable side effect.

In summary, we have demonstrated a strategy to simultaneously target pathogens and host inflammatory pathways in treating bacterial infections. To achieve this, we have generated lung targeting nanovesicles made from human neutrophils, and co-loaded RvD1 and CAZ inside the nanovesicles. The results show that neutrophil nanovesicles can specifically target inflamed mouse lungs and deliver RvD1 and CAZ to the infectious lesions. Delivery of RvD1 using neutrophil nanovesicles can accelerate the inflammation resolution and lower the antibiotics to treat infections. Our studies reveal a paradigm shift to develop nanoparticle-based therapeutics to treat bacterial infections by targeting infectious sites to control inflammation response and remove pathogens simultaneously.

## Materials and methods

### Materials

LPS (*Escherichia coli* 0111:B4), anthrone, dimethylsulfoxide (purity > 99.5%), NH_4_SCN, glucose, Histopaque 10771, and Histopaque 11191 were purchased from Millipore-Sigma (St. Louis, MO). Anti-Nup153 antibody, recombinant human and mouse TNF-α (carrier-free, purity 98%), Alexa Fluor@647 anti-mouse CD31 antibody, APC-anti-CD15, FITC-anti-CD11b antibody, and enzyme-linked immunosorbent assay (ELISA) kits for TNF-α, IL-1β, and IL-6 were bought from Biolegend (San Diego, CA). Lipid-staining dye 1,1′-Dioctadecyl-3,3,3′,3′-Tetramethylindotricarbocyanine Iodide (DiR), benzoxazolium, 3-octadecyl-2-[3-(3-octadecyl-2(3H)-benzoxazolylidene)-1-propenyl]-, perchlorate (DiO), 3H-Indolium, 2-(3-(1,3-dihydro-3,3-dimethyl-1-octadecyl-2H-indol-2-ylidene)-1-propenyl)-3,3-dimethyl-1-octadecyl-, perchlorate (DiL), and 4′,6-diamidino-2-phenylindole (DAPI) were purchased from Life Technologies (Grand Island, NY). Anti-ICAM-1, anti-integrin β_2_, and anti-GAPDH antibodies were purchased from Santa Cruz Biotechnology (Santa Cruz, CA). Pierce™BCA protein assay kit and ECL western blotting substrate were obtained from Thermo Fisher Scientific (Rockford, IL). The cell apoptosis detection kit was obtained from Invitrogen (Eugene, OR). CellTiter Aqueous One Solution Assay kit for cell growth was obtained from Promega (Madison, WI). RvD1 was from Cayman chemical company (Ann Arbor, MI). CAZ was bought from TCI Inc (Tokyo, Japan). L-*α*-phosphatidylcholine was obtained from Avanti Polar lipids (Alabaster, AL).

Bacterial strains *P. aeruginosa* PA103 was purchased from ATCC (Manassas, VA) and bioluminescent *P. aeruginosa* PAO1 strain was a gift from Harvard University. The strains were routinely cultured on sheep blood agar plates (Hardy Diagnostics, Santa Maria, CA) and amplified in LB medium in shaking flasks.

HUVECs were cultured in endothelium growth base media, supplemented with EGM SingleQuots (Lonza, Walkersville, MD). Cell lines, including HEK293T and NHF cells (gifts from Dr. Zhu’s lab at Washington State University) were grown in Dulbecco’s modified Eagle medium (Lonza, Walkersville, MD) supplemented with 10% FBS (Atlanta, Flowery Branch, GA) and 1% Pen Strep solution (Life Technologies, Grand Island, NY). Trypsin 0.25% solution and HBSS buffer (without Ca^2+^, Mg^2+^, and phenol red) was obtained from Corning (Corning, NY).

### Human neutrophil isolation

The protocol of human subjects has been approved by the Institutional Review Board, WSU, and the informed consent was obtained from all blood donors. The venous blood was collected through antecubital venipuncture from healthy adults (male or female) and placed in EDTA-K3 human blood collection tubes (BD Franklin Lakes, NJ). Isolation of human neutrophils from the whole blood was performed by the gradient density centrifugation approach using Histopaque 10771 and 11191 in 15 ml polypropylene centrifuge tubes. Briefly, 3 ml of Histopaque 10771 was carefully layered on top of 3 ml of Histopaque 11191 in a centrifuge tube. Subsequently, 6 ml of the collected blood was decanted on this discontinuous density gradient liquid. The tube was centrifuged at 890 g for 30 min at 20 °C. The neutrophils were carefully obtained after removing the top layers. The neutrophil suspension was diluted by two times in PBS without Ca^2+^ and Mg^2+^. The neutrophil suspension was then centrifuged at 870 g for 5 min at 4 °C. The supernatant was removed, and the cell pellet was resuspended in the RBC lysis buffer (BioVision, Milpitas, CA). The suspension buffer was left on bench for 30 min to lyse RBCs. The neutrophils were then pelleted and washed once with PBS. The purity of isolated neutrophils was identified by a confocal microscope (A1R plus, Nikon, Japan) and a flow cytometer (Gallios, Beckman, Gemany) after staining with DAPI or Alexa Fluor 488-anti-CD11b and APC-anti-CD15 monoclonal antibody. Separated human RBCs were also collected for preparation of RBCVs.

### Human neutrophil vesicle preparation and CAZ loading

Neutrophils were resuspended in PBS containing 10% human plasma and incubated at 37 °C for 1 h after LPS was added at a final concentration of 100 μg/ml to activate neutrophils. The cells were then pelleted and resuspended in HBSS at 1–5 × 10^6^/ml. The suspension solution with a volume around 10 ml was placed in a nitrogen cavitation chamber to disrupt the cells, followed by the ultrafast centrifugation to obtain neutrophil membrane-formed nanovesicles (NVs)^22,23^. To make CAZ-loaded NVs, 20 mg/ml CAZ was added in the cell suspension and then the cell suspension was placed in a nitrogen cavitation chamber to generate nanovesicles. Sizes and surface charges of nanovesicles were measured using either qNano particle analyzer (Izon science, Cambridge, MA) or Mavern Zetasizer Nano ZS90 (Westborough, MA). RBC nanovesicles (RBCVs) were prepared following the same procedure of making neutrophil nanovesicles.

### RvD1 loading of NVs and RBCVs

RvD1 and prewarmed NVs or RBCVs (1 : 400, w/w) were incubated in HBSS (pH adjusted to 5) at 37 °C for 40 min. The vesicles were then pelleted and washed once with HBSS. To determine the loading efficiency, the loaded RvD1 was extracted with ethanol and then RvD1 concentrations were measured using HPLC-mass spectroscopy.

### Molecular compositions of nanovesicles

To determine the molecular compositions of nanovesicles, we measured proteins, phospholipids and carbohydrate and calculated their percentages in the total mass of nanovesicles. To quantify proteins in nanovesicles, we used bicinchoninic acid assay (BCA) assays. To measure the phospholipids in nanovesicles, the reaction solution was prepared using 0.27 g FeCl_3_·6H_2_O and 0.3 g NH_4_SCN in 10 ml distilled water. The chloroform-extracted phospholipid was mixed with the reaction solution at a ratio of 2:1 (v/v), followed by thoroughly mixing. The mixtures were placed on the bench to allow the formation of two layers. The OD_488_ values of lower layer was measured. 1 mg/ml L-*α*-phosphatidylcholine in chloroform was used as a reference^46,47^. To measure the carbohydrate contents in nanovesicles, the reaction solution was prepared using 100 mg anthrone in 100 ml at 14 M H_2_SO_4_. Nanovesicles (prepared in PBS) or the serially diluted standard solutions were mixed with 5-fold volume of reaction solution, followed by incubating in a boiling water bath for 10 min. The mixture solution was cooled down and measured on a colorimeter at OD_625_. 1 mg/ml D-glucose in PBS was used as a reference of carbohydrate^48^.

### Western blottings

The proteins of NVs were quantified using western blottings and imaged by Bio-Rad Molecular imager (ChemiDoc XRS, Bio-Rad, Hercules, CA). The antibodies of integrin β_2_, Nup153, and GAPDH (Santa Cruz Biotechnologies, Santa Cruz, CA) were used. For the inhibiting effect on the ICAM-1 expression by RvD1, the monoclonal anti-ICAM-1 antibody was used.

### CAZ and RvD1 measurement

CAZ in nanovesicles was determined using a Waters 2690 HPLC system (Waters, Milford, MA), with mobile phase using methanol: 20 mM KH_2_PO_4_ pH 3.5 = 25 : 75 at 1 ml/min. The separating column was Restek C18 25 cm × 4.6 μm. The signal was collected at 260 nm. The RvD1 quantitation was done using an Acquity I-Class Ultra pressure liquid chromatography system, containing a PDA eλ detector, an Xevo G2-S QTof mass detector and a binary solvent manager (Waters, Milford, MA), with a C18 column 100 × 2.1 mm. The column temperature was set at 50 °C. The flow phase contained a solvent A (water with NH_4_F 5 mM and formic acid 2 mM) and a solvent B (acetonitrile). The flow phase was initially set at 35% B for 0.5 min, then increased to 100% in 3 min, and finally maintained at 100% B for 2 min. UV absorption wavelength at 301 nm was used to monitor signal of RvD1. Flow speed was maintained at 0.5 ml/min. The peak of RvD1 was verified by mass spectrum.

### Animals

All experiments using animal models were approved by the Institutional Animal Care and Use Committee of Washington State University, USA. Male CD-1, 6–8 weeks, mice were purchased from Envigo (Denver, CO) and housed in a SPF-grade facility with normal food and drink.

### Pharmacokinetics, biodistribution of NVs and IVIS analysis

NVs or RBCVs were labeled by lipid dye DiR by directly adding the 0.2% (w/w) dye into the nanovesicle solutions, followed by vigorous agitation and incubation for 20 min at 37 °C for 20 min. The nanovesicles were i.v. administered to mice. At the indicated time points, the blood was collected via facial blood sampling. DIR in the plasma was extracted with a ninefold volume of pure ethanol and the DiR contents were measured using a spectrometer based on calibration of the standard DIR. To determine the deposition of nanovesicles in the mouse lungs, the lungs were collected and imaged using an IVIS imaging system (Caliper Life Sciences, Hopkinton, MA).

### Intravital microscope imaging

TNF-α (500 ng in 250 μl saline) was intra-scrotally injected into a mouse to cause vascular inflammation. Three hours after TNF-α injection, the mouse was anesthetized with ketamine (100 mg/kg)/xylazine (5 mg/kg) and maintained at 37 °C on a thermo-controlled rodent blanket. A tracheal tube was inserted and a right jugular vein was cannulated for infusion of nanovesicles or antibodies. After the scrotum was incised, the testicle and surrounding cremaster muscles were exteriorized onto an intravital microscopy tray. The cremaster preparation was perfused with thermo-controlled (37 °C) and aerated (95% N_2_, 5% CO_2_) bicarbonate-buffered saline throughout the experiment. Images were recorded using a Nikon A1R + laser scanning confocal microscope with a resonant scanner. We simultaneously infused with DiO-labeled RBCVs (DiO-RBCVs) and DiL-labeled NVs (DiL-NVs) (0.1 mg/mouse) at the same concentrations into the TNF-α-treated or normal mouse, water-immersion objective with NA = 1.1 was used to image cremaster venules. The images were taken simultaneously at 488 nm for DiO-labeling and 560 nm for DiL-labeling^22^.

### NVs bind to endothelium

HUVECs (2 × 10^5^/well) were seeded on a cover slip in a 3.5 cm dish, containing a glass slip, and activated for 4 h using 50 ng/ml TNF-α. Thereafter, one group of wells were supplemented with 10 μg/ml anti-ICAM-1 antibody and incubated for 30 min to block the interactions between integrin β_2_ and ICAM-1. The medium in all the wells was replaced with a fresh medium, containing 5 μg DiO-NVs vesicles. The cells were further cultured for 1 h under continuous agitation to mimic in vivo conditions. To quantitatively analyze the binding of nanovesicles to HUVECs and their uptake, at an equal fluorescence intensity DiO-RBCVs incubated with activated HUVECs under the same conditions. After incubation, cells were then washed twice with PBS and fixed with 4% PFA for 10 min on ice. After washing twice with PBS, the cells were mounted on a slide with a mounting reagent containing DAPI and imaged using a confocal microscope.

### Cell toxicity assay

One day before the assay, 10,000/well HUVECs, HEK293T, and NHF cells were seeded in 96-well plates. Then, the medium of the cells was changed to 200 μl individual media containing different concentrations of NVs. The cells were further cultured in a cell incubator. Twenty hours later, 20 μl cell titer 3-(4,5-dimethylthiazol-2-yl)-5-(3-carboxymethoxyphenyl)-2-(4-sulfophenyl)-2H-tetrazolium stock solution was added into each well. Four hours later, the wells were measured using a plate reader at OD_490_ (SynergyOne, BioTek, Winooski, VT) to assess the cell viability.

### Enhanced therapeutical effect of NVs in ALI mouse model

Mice were randomly separated into three groups. The mice were intratracheally (i.t.) given LPS 5 mg/kg and then HBSS, RvD1-free drug (67 ng/animal), and RvD1-NVs (67 ng/animal in RvD1) was i.v. administered respectively 2 h later. At the indicated time points, the animals were anesthetized and BALFs were collected. The total cells in each fluid sample were counted using a hemocytometer under a microscope. The ratios of neutrophils and macrophage were determined by flow cytometry after staining with Ly-6G and F4/80 monoclonal antibodies. The protein contents in BALFs were measured using a BCA kit. The cytokine levels were determined using ELISA kits. The experiments were all performed in triplicate.

Resolution indices were determined. *T*_max_ represents the time point when PMN number reaches the maximum; *T*_50_ is the time point when PMN numbers reduce to 50% of the maximum; *R*_i_ (resolution interval) is calculated by *T*_50_ − *T*_max_, a time period when 50% PMNs are lost^30^.

### Histology assay

Animals were i.t. challenged by 5 mg/kg LPS, at 2 h later followed by different treatments including HBSS, free RvD1, and RvD1-NVs. Twenty hours post treatments, mice were killed by carbon dioxide asphyxiation and lungs were removed and quickly frozen on dry ice in tissue freezing medium. The tissues were sectioned at 5 μm and stained with hematoxylin and eosin for pathology. The slides were imaged under a microscope (ZEISS, Axio Observer Z1, Gemany).

### RvD1 inhibited the expression of ICAM-1 on HUVECs

Approximately 40,000 HUVECs were seeded in a well on a 12-well plate 1 day prior the experiment. Sixteen hours later, the cells were changed to the culture medium with or without TNF-α (50 ng/ml) and RvD1 (33 ng/ml, free or loaded in NVs). Four hours later, the cells were collected and lysed for analysis of ICAM-1 expression using western blottings.

### Apoptosis assay

After treated with LPS (100 μg/ml), LPS/RvD1 (33 ng/ml) or LPS/RvD1-NVs (RvD1, 33 ng/ml), the apoptosis of human neutrophils was analyzed using a cell apoptosis kit. Briefly, neutrophils were suspended in the binding buffer and the reagent Alexa Fluor 488–Annexin V was added and incubated for 15 min. Then propidium iodide was added, followed by flow cytometry analysis. The experiment was performed in triplicate.

### CAZ-NVs inhibited *P. aeruginosa* growth in infection mouse model

Bioluminescent *P. aeruginosa* PAO1-luc strain was cultured in brain heart infusion media plates and collected in PBS. After titer measurement, 10^5^ colony forming unit (CFU) bacteria in 50 μl PBS was intratracheally injected into the lungs of CD-1 mice. Four hours later, the animals were administered with PBS, free CAZ (2 mg/kg), or CAZ-NVs (CAZ, 2 mg/kg). At 0, 12, and 24 h after treatment, the bioluminescence of animals was imaged using IVIS imaging system. The lungs and plasma were sampled after the last imaging and homogenized to measure the CFUs and cytokines, respectively.

### CAZ-RvD1-NVs enhanced therapies of bacterial infections

CD-1 mice were inoculated with *P. aeruginosa* PA103 (5 × 10^5^ CFUs, intratracheal) and, 4 h later, the vehicle, free RvD1, free CAZ, RvD1 + CAZ, RvD1-CAZ-RBCVs, and RvD1-CAZ-NVs were i.v. given to the animals (67 ng/animal for RvD1, 1 mg/kg for CAZ). At 0, 12, and 24 h after treatment, blood and peritoneal exudates were collected. *P. aeruginosa* titers and cytokine levels in blood and BALFs were measured by LB plate culture and ELISA, respectively.

### Bacterium phagocytosis by macrophages

The mouse peritoneal macrophages were isolated and cultured overnight in RPMI1640 medium supplemented with 20% FBS. The adherent macrophages were digested and utilized for the assay. *P. aeruginosa* were opsonized with 10% mouse serum and stained with lipid dye DiO before the phagocytosis assay. Bacteria (2 × 10^6^ CFU) were incubated with 10^5^ macrophages with or without 33 ng/ml RvD1 and 10 μg/ml CAZ at 37 °C for 60 min. The macrophages were then washed once with PBS and subject to analysis by flow cytometry.

### Statistical and reproducibility

All data are expressed as means ± SD. Differences between groups were examined for statistical significance with One-way analysis of ariance by GraphPad Prism 5.0. Differences at *P* < 0.05, *P* < 0.01, *P* < 0.001 are considered significant, very significant, and extremely significant, respectively. In addition, the sample sizes and number of replicates were described in the text or figure captions.

### Reporting summary

Further information on research design is available in the Nature Research Reporting Summary linked to this article.

## Supplementary information

Supplementary Information

Description of Additional Supplementary Files

Supplementary Data 1

Reporting Summary

## Data Availability

Source data for all graphs in this article are included in Supplementary Data. Uncropped data for all blots in this article are included in Supplementary Information. The information and data in this article are available from the corresponding author on request.

## References

[1] Morand A, Morand JJ (2017). *Pseudomonas aeruginosa* in dermatology. Ann. Dermatol Vener..

[2] Lee CH (2017). Risk factors and clinical significance of bacteremia caused by *Pseudomonas aeruginosa* resistant only to carbapenems. J. Microbiol. Immunol. Infect..

[3] Carmeli Y (2016). Ceftazidime-avibactam or best available therapy in patients with ceftazidime-resistant Enterobacteriaceae and *Pseudomonas aeruginosa* complicated urinary tract infections or complicated intra-abdominal infections (REPRISE): a randomised, pathogen-directed, phase 3 study. Lancet Infect. Dis..

[4] Sadikot RT (2006). Targeted immunomodulation of the NF-kappaB pathway in airway epithelium impacts host defense against *Pseudomonas aeruginosa*. J. Immunol..

[5] Mizgerd JP (2012). Respiratory infection and the impact of pulmonary immunity on lung health and disease. Am. J. Respir. Crit. Care Med..

[6] Matthay MA, Ware LB, Zimmerman GA (2012). The acute respiratory distress syndrome. J. Clin. Invest..

[7] Matthay MA, Zemans RL (2011). The acute respiratory distress syndrome: pathogenesis and treatment. Annu. Rev. Pathol..

[8] Ware LB, Matthay MA (2005). Clinical practice. Acute pulmonary edema. N. Engl. J. Med.

[9] Thompson BT, Chambers RC, Liu KD (2017). Acute respiratory distress syndrome. N. Engl. J. Med..

[10] Roope, L. S. J. et al. The challenge of antimicrobial resistance: what economics can contribute. *Science* **364**, 10.1126/science.aau4679 (2019). 10.1126/science.aau4679 30948524

[11] Aarestrup FM, Engberg J (2001). Antimicrobial resistance of thermophilic Campylobacter. Vet. Res.

[12] Kashef N, Hamblin MR (2017). Can microbial cells develop resistance to oxidative stress in antimicrobial photodynamic inactivation?. Drug Resist. Updat..

[13] Dong, X. Y., Zhang, C. Y., Gao, J. & Wang, Z. J. Targeting of nanotherapeutics to infection sites for antimicrobial therapy. *Adv. Ther. Germany* **2**, UNSP 1900095 10.1002/adtp.201900095 (2019). 10.1002/adtp.201900095 PMC7731920 33313384

[14] Hussain S (2018). Antibiotic-loaded nanoparticles targeted to the site of infection enhance antibacterial efficacy. Nat. Biomed. Eng..

[15] Zhang CY, Gao J, Wang Z (2018). Bioresponsive nanoparticles targeted to infectious microenvironments for sepsis management. Adv. Mater..

[16] Zhang CY (2019). pH-responsive nanoparticles targeted to lungs for improved therapy of acute lung inflammation/injury. ACS Appl. Mater. Interfaces.

[17] Zhang CY (2019). Nanoparticle-induced neutrophil apoptosis increases survival in sepsis and alleviates neurological damage in stroke. Sci. Adv..

[18] Tabas I, Glass CK (2013). Anti-inflammatory therapy in chronic disease: challenges and opportunities. Science.

[19] Phillipson M, Kubes P (2011). The neutrophil in vascular inflammation. Nat. Med..

[20] Wagner DD, Frenette PS (2008). The vessel wall and its interactions. Blood.

[21] Wang ZJ, Li J, Cho J, Malik AB (2014). Prevention of vascular inflammation by nanoparticle targeting of adherent neutrophils. Nat. Nanotechnol..

[22] Gao J, Chu DF, Wang ZJ (2016). Cell membrane-formed nanovesicles for disease-targeted delivery. J. Control Release.

[23] Gao J, Wang SH, Wang ZJ (2017). High yield, scalable and remotely drug-loaded neutrophil-derived extracellular vesicles (EVs) for anti-inflammation therapy. Biomaterials.

[24] Dong, X. et al. Neutrophil membrane-derived nanovesicles alleviate inflammation to protect mouse brain injury from ischemic stroke. *ACS Nano*, 10.1021/acsnano.8b06572 (2019). 10.1021/acsnano.8b06572 PMC6424134 30673266

[25] Serhan CN (2002). Resolvins: a family of bioactive products of omega-3 fatty acid transformation circuits initiated by aspirin treatment that counter proinflammation signals. J. Exp. Med..

[26] Oh SF, Pillai PS, Recchiuti A, Yang R, Serhan CN (2011). Pro-resolving actions and stereoselective biosynthesis of 18S E-series resolvins in human leukocytes and murine inflammation. J. Clin. Invest..

[27] Spite M (2009). Resolvin D2 is a potent regulator of leukocytes and controls microbial sepsis. Nature.

[28] Krishnamoorthy S, Recchiuti A, Chiang N, Fredman G, Serhan CN (2012). Resolvin D1 receptor stereoselectivity and regulation of inflammation and proresolving microRNAs. Am. J. Pathol..

[29] Krishnamoorthy S (2010). Resolvin D1 binds human phagocytes with evidence for proresolving receptors. Proc. Natl Acad. Sci. USA.

[30] Chiang N (2012). Infection regulates pro-resolving mediators that lower antibiotic requirements. Nature.

[31] Alexis F, Pridgen E, Molnar LK, Farokhzad OC (2008). Factors affecting the clearance and biodistribution of polymeric nanoparticles. Mol. Pharmaceutics.

[32] Zhang X (2017). Remote loading of small-molecule therapeutics into cholesterol-enriched cell-membrane-derived vesicles. Angew. Chem..

[33] Fang RH, Jiang Y, Fang JC, Zhang L (2017). Cell membrane-derived nanomaterials for biomedical applications. Biomaterials.

[34] Kolaczkowska E, Kubes P (2013). Neutrophil recruitment and function in health and inflammation. Nat. Rev. Immunol..

[35] Freitas M, Porto G, Lima JL, Fernandes E (2008). Isolation and activation of human neutrophils in vitro. The importance of the anticoagulant used during blood collection. Clin. Biochem..

[36] Fermino ML (2011). LPS-induced galectin-3 oligomerization results in enhancement of neutrophil activation. PLoS ONE.

[37] Briegel A (2009). Universal architecture of bacterial chemoreceptor arrays. Proc. Natl Acad. Sci. USA.

[38] Chu D, Dong X, Shi X, Zhang C, Wang Z (2018). Neutrophil-based drug delivery systems. Adv. Mater..

[39] Ley K, Laudanna C, Cybulsky MI, Nourshargh S (2007). Getting to the site of inflammation: the leukocyte adhesion cascade updated. Nat. Rev. Immunol..

[40] Nourshargh S, Alon R (2014). Leukocyte migration into inflamed tissues. Immunity.

[41] Brown KA (2006). Neutrophils in development of multiple organ failure in sepsis. Lancet.

[42] Nathan C (2006). Neutrophils and immunity: challenges and opportunities. Nat. Rev. Immunol..

[43] Serhan CN (2007). Resolution phase of inflammation: novel endogenous anti-inflammatory and proresolving lipid mediators and pathways. Annu. Rev. Immunol..

[44] Seki H (2010). The anti-inflammatory and proresolving mediator resolvin E1 protects mice from bacterial pneumonia and acute lung injury. J. Immunol..

[45] Serhan CN, Chiang N (2013). Resolution phase lipid mediators of inflammation: agonists of resolution. Curr. Opin. Pharmacol..

[46] Stewart JC (1980). Colorimetric determination of phospholipids with ammonium ferrothiocyanate. Anal. Biochem..

[47] Albrink MJ, Man EB (1959). Serum triglycerides in coronary artery disease. AMA Arch. Intern. Med..

[48] Trevelyan WE, Forrest RS, Harrison JS (1952). Determination of yeast carbohydrates with the anthrone reagent. Nature.

