# Supplementary material for: Co-delivery of resolvin D1 and antibiotics with nanovesicles to lungs resolves inflammation and clears bacteria in mice
Source: Commun Biol. 2020 Nov 16;3:680. doi: 10.1038/s42003-020-01410-5 (PMC7669882; doi:10.1038/s42003-020-01410-5)
Supplement: Supplementary file 1 — Supplementary Information [file 42003_2020_1410_MOESM1_ESM.docx]

**Supplementary Information**

**Co-delivery of Resolvin D1 and Antibiotics with Nanovesicles to Lungs Resolves Inflammation and Clears Bacteria in Mice**

Jin Gao^1^, Sihan Wang^1^, Xinyue Dong^1^, Leon G. Leanse^2^, Tianhong Dai^2^,

Zhenjia Wang^1,^*

1, Department of Pharmaceutical Sciences, College of Pharmacy and Pharmaceutical Sciences, Washington State University, Spokane, WA 99202, USA

2, Wellman Center for Photomedicine, Massachusetts General Hospital, Harvard Medical School, Boston, MA, United States

*Correspondence should be addressed to [zhenjia.wang@wsu.edu](mailto:zhenjia.wang@wsu.edu)

**This file includes:**

Figure S1. Isolation of human neutrophils and RBCs.

Figure S2. Identification and activation of the isolated neutrophils.

Figure S3. Uncropped blots of Fig S2D.

Figure S4. Uncropped blots of Figure 2D.

Figure S5. Characterization of RBCVs.

Figure S6 Uncropped blot of Figure S5C.

Figure S7. Biodistribution of NVs in mice with lung inflammation.

Figure S8. Toxicity of NVs on HUVECs, NHF cells and 293T cells.

Figure S9. RvD1loaded in NVs measured by UPLC.

Figure S10. Size and surface measurement of NVs and RvD1-NVs.

Figure S11. Uncropped blots of Figure 4I.

Figure S12. The bioluminescent imaging of P. aeruginosa PAO1-Luc strain.

Figure S13. CAZ in formulations measured by HPLC.

Figure S14. CAZ release profiles from CAZ-NVs measured by HPLC.

Figure S15. Size and surface charge measurements of NVs, CAZ-NVs and RvD1-CAZ-NVs.


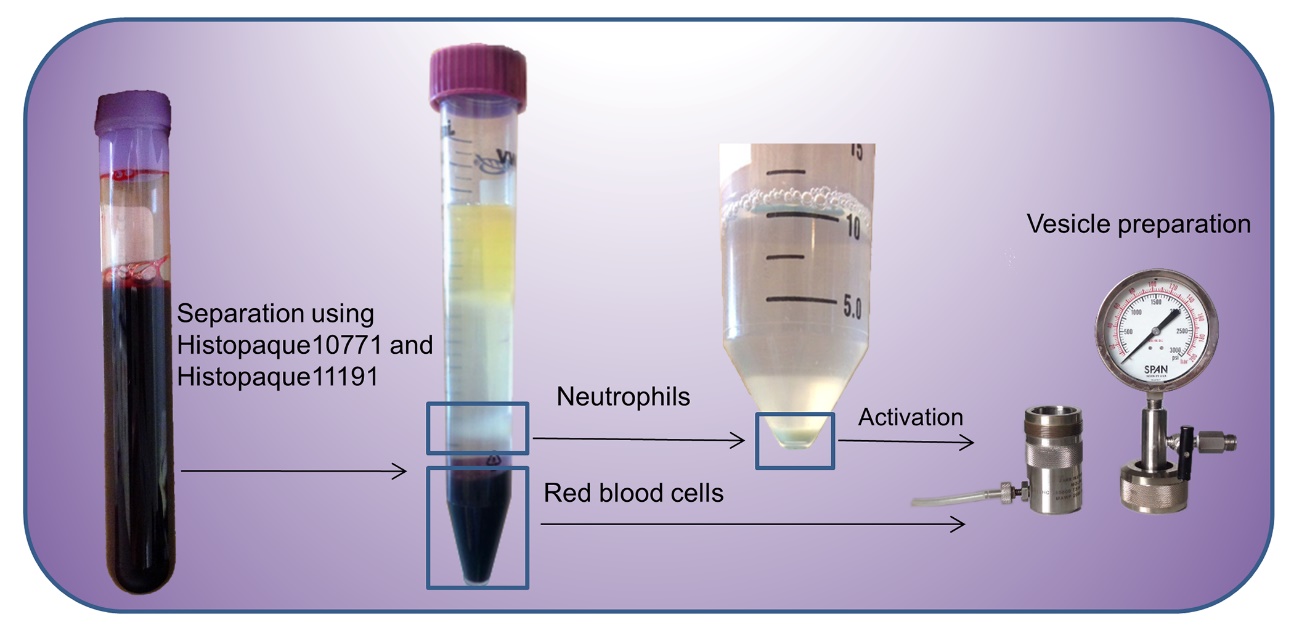


Figure S1. Isolation of human neutrophils and RBCs for generation of cell membrane-derived nanovesicles. The fresh human blood was collected from healthy adult donors. Blood cells in Histopaque10771 and Histopaque11191 reagents were separated using the gradient density centrifugation. Neutrophils between the two reagents were collected and washed, and then they were activated with LPS (100 μg/ml in the presence of 20% human plasma) for 60 min. Finally, activated cells were used to generate neutrophil nanovesicles using the nitrogen cavitation approach (the far-right photo is a nitrogen cavitation chamber). RBC nanovesicles were produced similarly.


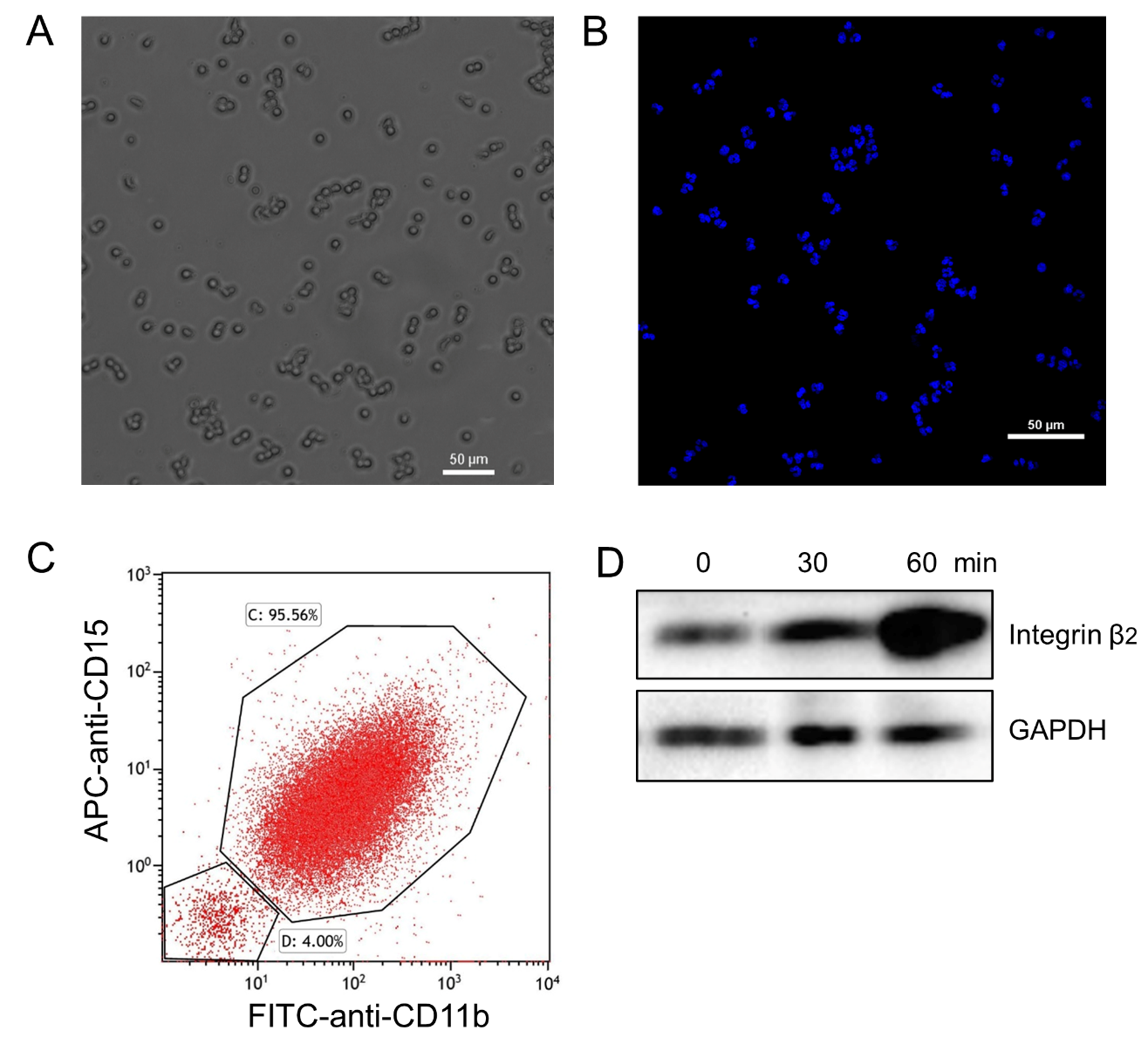


Figure S2. Identification and activation of isolated neutrophils. (A) Cell image under a microscope. (B) Cell image was taken by a confocal microscope after DAPI staining. (C) Purity of neutrophils measured via flow cytometry after staining with APC-anti-CD15 and FITC-anti-CD11b. D: Integrin β_2_ was upregulated after the treatment with LPS at 100 μg/ml for 1 h.


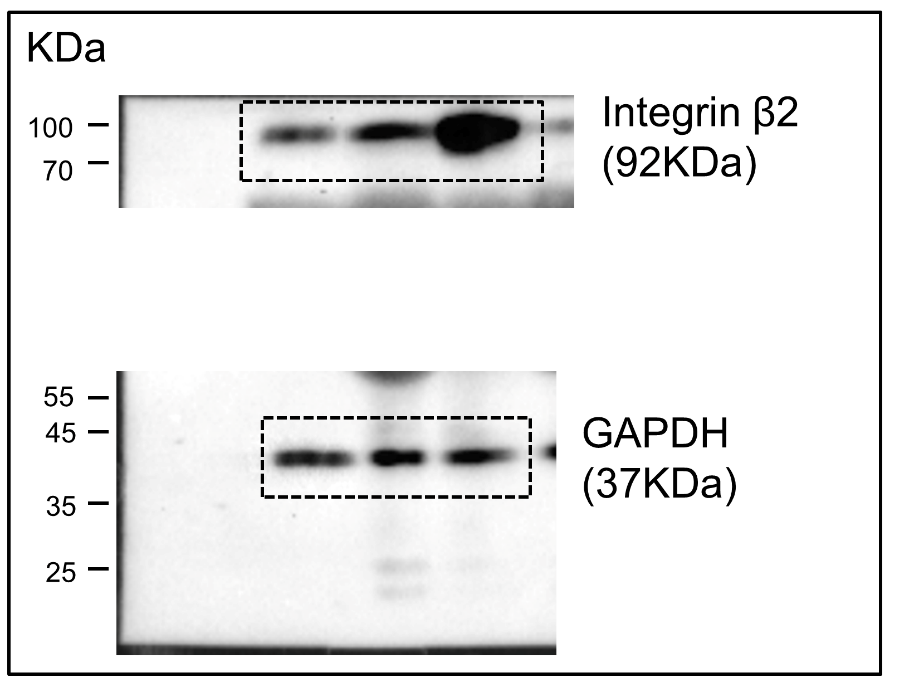


Figure S3. Uncropped blots of Fig S2D.


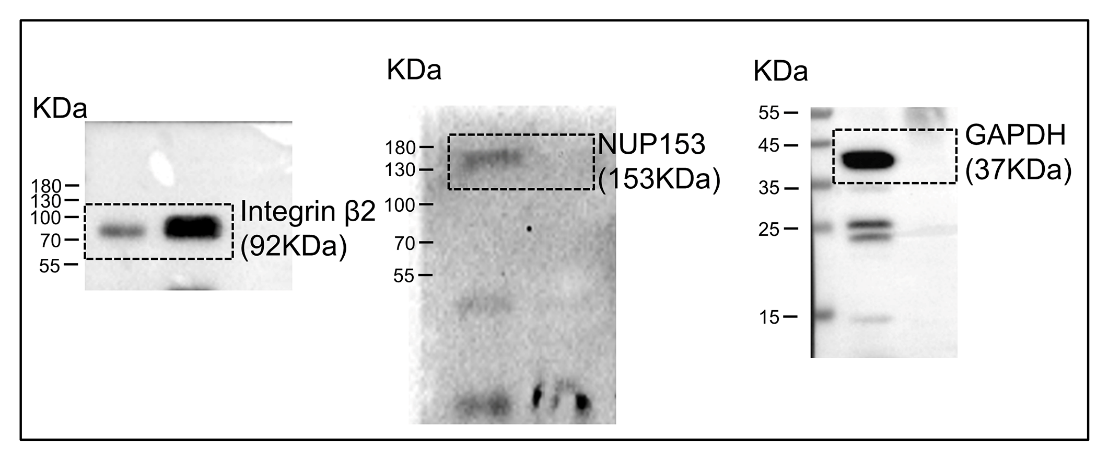


Figure S4. Uncropped blots of Figure 2D.


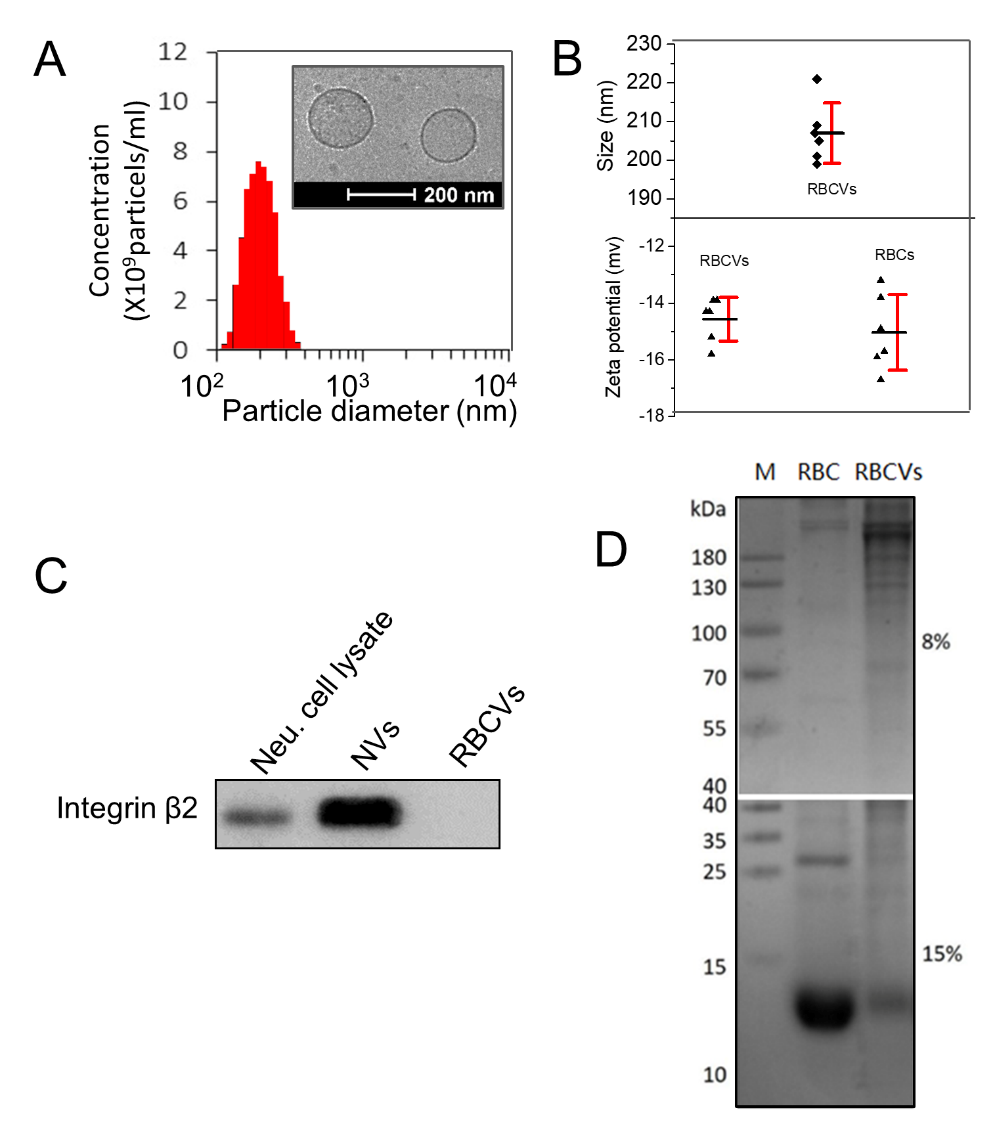


Figure S5. Characterization of RBCVs. (A) Size of RBCVs measured by DLS and Cryo-TEM (inset). (B) Mean size and surface charges of RBCVs and RBCs measured by DLS. (C) Western blots of integrin β_2_ expression on neutrophils, NVs and RBCVs. (D) Protein profiles of RBCs and RBCVs on 8% and 15% SDS-PAGE at the same amount of protein loading. Data are presented as the mean ± SD, n=6 independent samples.


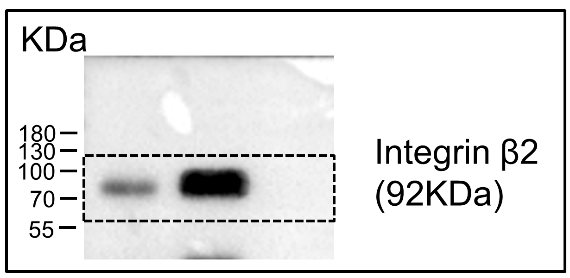


Figure S6 Uncropped blot of Figure S5C.


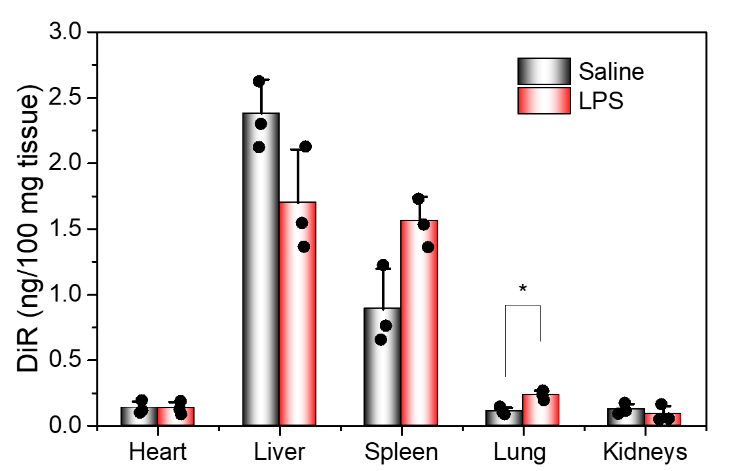


Figure S7. Biodistribution of NVs in mice with lung inflammation. The biodistribution of NVs was measured at 2 h after i.v. injection of NVs. A standard curve of DiR was established to calculate the concentrations of homogenization solution of tissues. The wavelength was set at 750ex/780em. Data are presented as the mean ± SD, n=3 biologically independent animals. *P < 0.05.


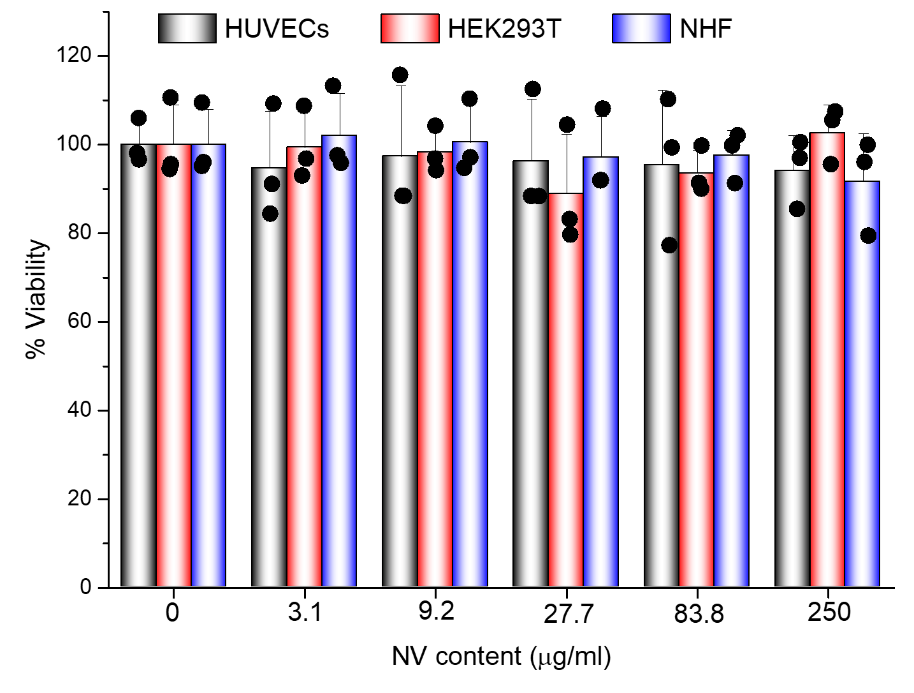


Figure S8. Toxicity of NVs on HUVECs, NHF cells and 293T cells 24 h after incubation of NVs with the cells. Data are presented as the mean ± SD, n=3 independent experiments.


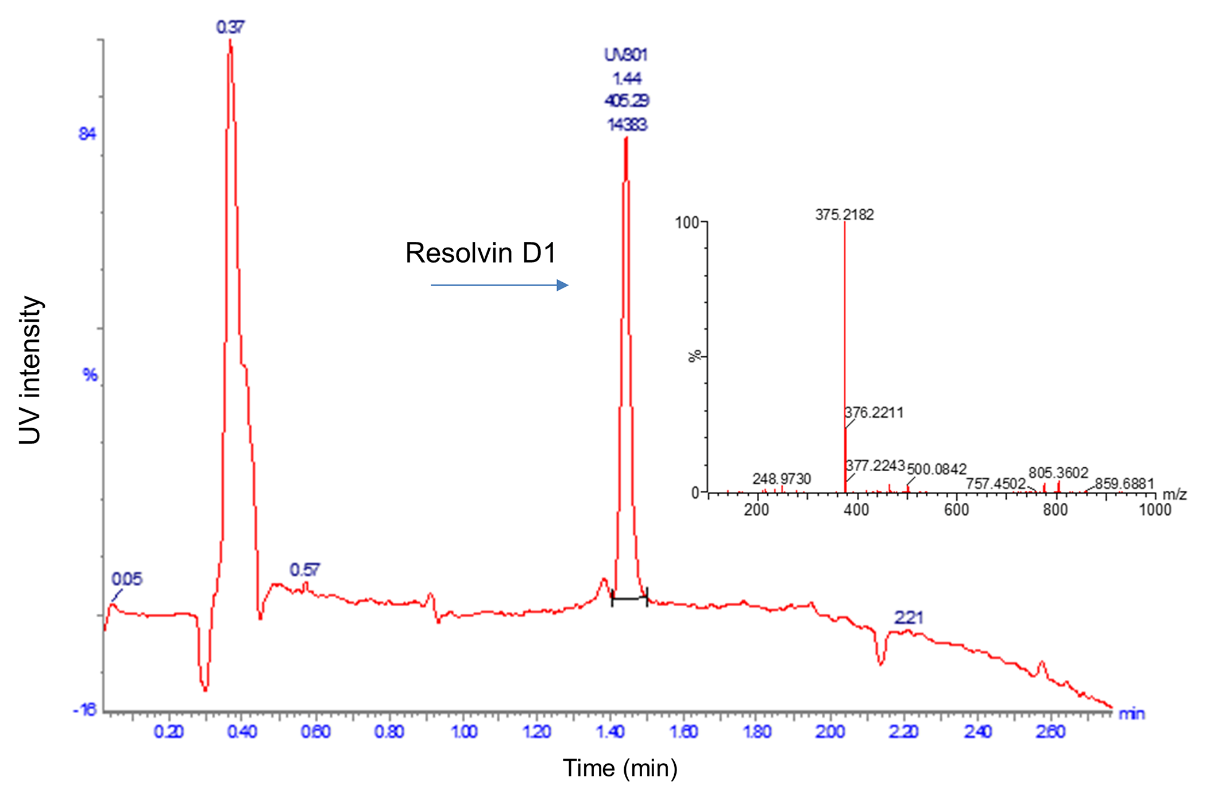


Figure S9. RvD1loaded in NVs measured by UPLC. A C18 column 100×2.1 mm was used. Column temperature was set at 50^o^C. The flow phase contained solvent A: ammonium fluoride (5 mM) and formic acid (2mM) in water and solvent B: acetonitrile. UV absorption wavelength at 301 nm was used to monitor signal of RvD1. Flow speed was maintained at 0.5 ml/min. The mass of RvD1 was also verified by mass spectrum (Inset).


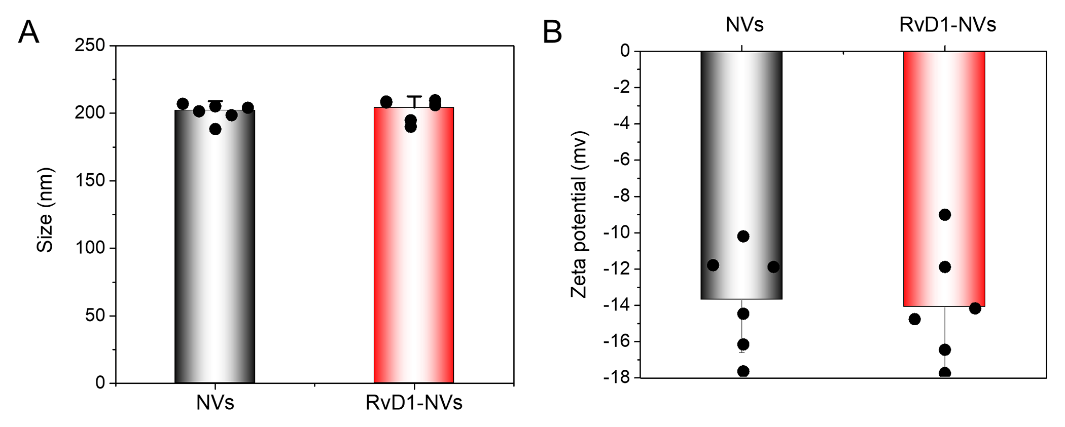


Figure S10. Sizes (A) and surface charges (B) of NVs and RvD1-NVs measured using Malvern Zetasizer Nano ZS90. Data are presented as the mean ± SD, n=6 independent samples.


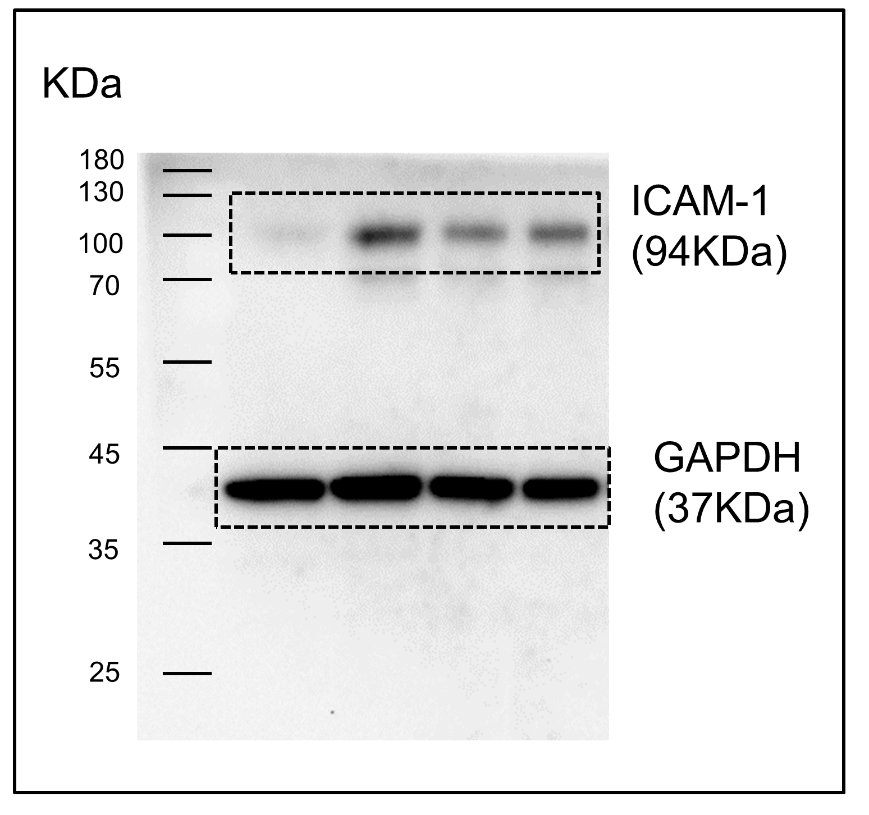


Figure S11. Uncropped blots of Figure 4I.


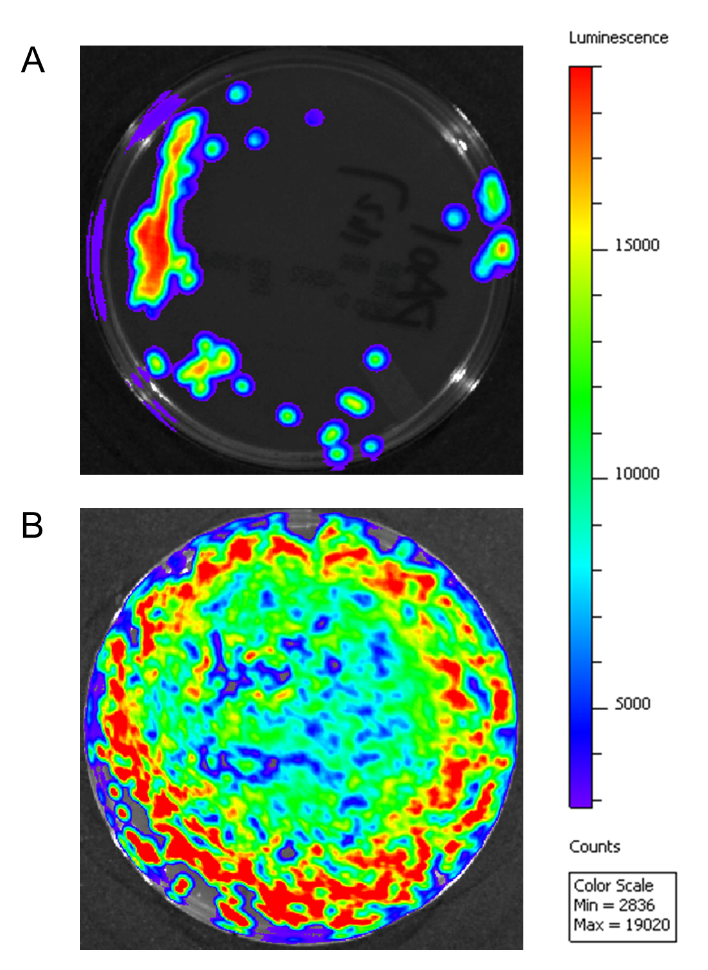


Figure S12. Bioluminescent images of *P. aeruginosa* *PAO1-Luc* strain (A) and in BALFs collected from the mouse lung infected by *P. aeruginosa* *PAO1-Luc*.


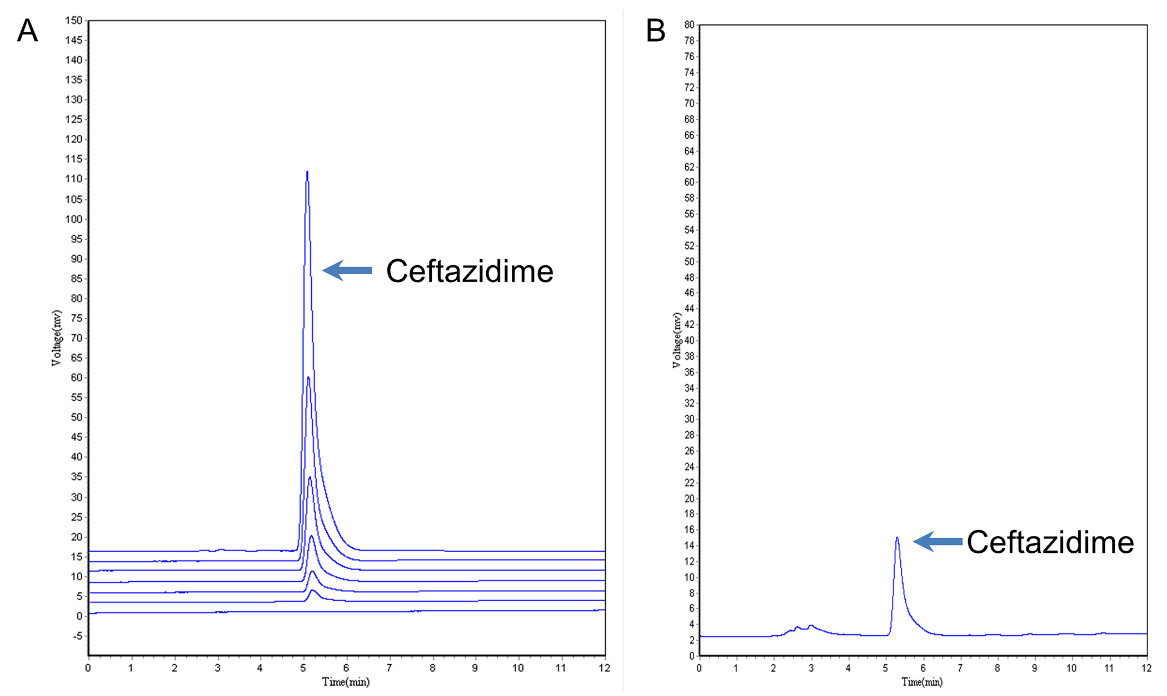


Figure S13. CAZ inside NVs was measured by HPLC. The mobile phase was methanol/monopotassium phosphate (20 mM at pH3.5) = 25/75 at 1ml/min. The column was Restek C18 25 cm×4.6 μm. The wavelength was monitored at 260 nm. Standard curves at different concentrations of CAZ (A), and CAZ-NVs was measured (B).


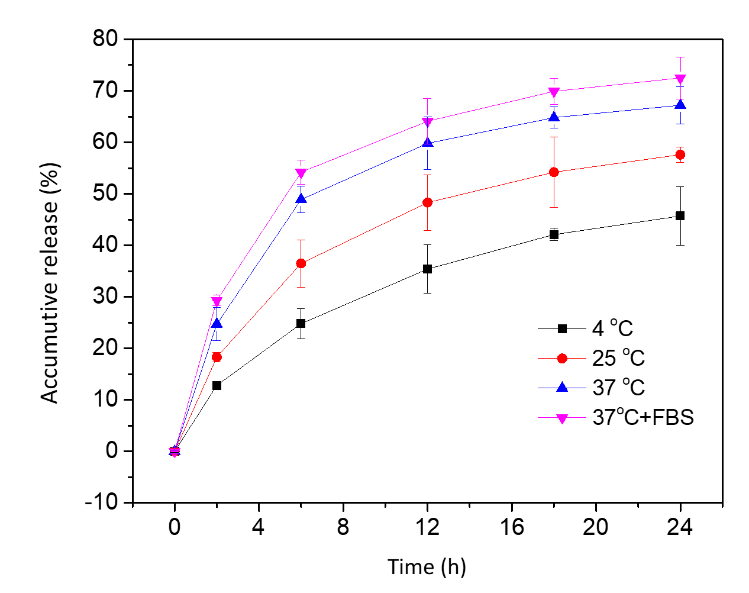


Figure S14. CAZ release profiles from CAZ-NVs by HPLC. The mobile phase was methanol/monopotassium phosphate (20 mM at pH3.5) = 25/75 at 1ml/min. The column was Restek C18 25 cm×4.6 μm. The wavelength was monitored at 260 nm. Data are presented as the mean ± SD, n=3 independent samples.


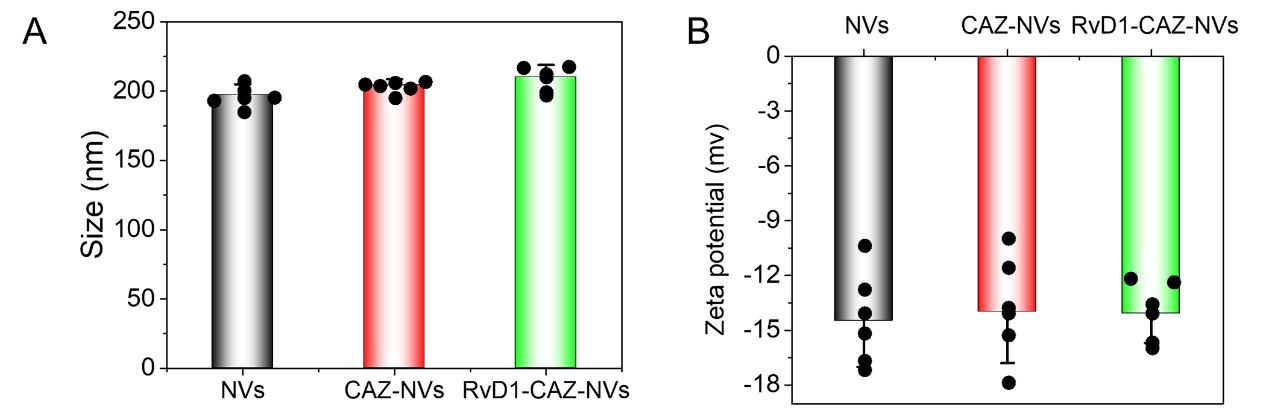


Figure S15. Sizes (A) and surface charges (B) of NVs, CAZ-NVs and RvD1-CAZ-NVs were measured by DLS. Data are presented as the mean ± SD, n=6 independent samples.
