# Supplementary material for: Co-delivery of resolvin D1 and antibiotics with nanovesicles to lungs resolves inflammation and clears bacteria in mice
Source: Commun Biol. 2020 Nov 16;3:680. doi: 10.1038/s42003-020-01410-5 (PMC7669882; doi:10.1038/s42003-020-01410-5)
Supplement: Supplementary file 2 — Description of Additional Supplementary Files [file 42003_2020_1410_MOESM2_ESM.pdf]

Description of Additional Supplementary Files

File Name: Supplementary Data 1
Description:
Fig 2B Sizes and zeta potentials of NVs and neutrophils
Fig 2E Components of NVs
Fig 3D Photons of lung tissues by IVIS
Fig 3E Biodistribution of NVs by fluor spectrometry
Fig 3F Pharmacokinetics of NVs and RBCVs
Fig 4B Neutrophil count in lungs
Fig 4C Proteins in BALFs
Fig 4E TNF-α in BALFs
Fig 4F IL-1β in BALFs
Fig 4G IL-6 in BALFs
Fig 4H IL-2 in BALFs
Fig 4I ICAM-1 expression measurement by WB
Fig 4J Apoptosis ratio assay
Fig 5C Photons by IVIS
Fig 5E Tissue CFU measurement
Fig 5F Cytokine measurement 2
Fig 6C CFU measurement in blood
Fig 6D Cytokines in blood
Fig 6E Cytokines in BALFs
Fig 6F Cell count in BALFs
Fig 6G Cell phagocytosis assay
Fig S5B Sizes and zeta potentials of RBCVs and RBCs
Fig S7 Biodistribution of NVs in organs
Fig S8 Cytotoxicity of NVs
Fig S10A Size of NVs and RvD1-NVs
FigS10B Zeta potentials of NVs and RvD1-NVs
Fig S14 CAZ release
Fig S15A Size measurement
Fig S15B Zeta potential measurement
