# Supplementary material for: Co-delivery of resolvin D1 and antibiotics with nanovesicles to lungs resolves inflammation and clears bacteria in mice
Source: Commun Biol. 2020 Nov 16;3:680. doi: 10.1038/s42003-020-01410-5 (PMC7669882; doi:10.1038/s42003-020-01410-5)
Supplement: Supplementary file 4 — Reporting Summary [file 42003_2020_1410_MOESM4_ESM.pdf]

nature research

Corresponding author(s): Zhenjia Wang

Last updated by author(s): Oct 10, 2020

Reporting Summary

Nature Research wishes to improve the reproducibility of the work that we publish. This form provides structure for consistency and transparency in reporting. For further information on Nature Research policies, see our Editorial Policies and the Editorial Policy Checklist.

Statistics

For all statistical analyses, confirm that the following items are present in the figure legend, table legend, main text, or Methods section.

n/a	Confirmed	
☐	☒	The exact sample size (*n*) for each experimental group/condition, given as a discrete number and unit of measurement
☐	☒	A statement on whether measurements were taken from distinct samples or whether the same sample was measured repeatedly
☐	☒	The statistical test(s) used AND whether they are one- or two-sided<br>*Only common tests should be described solely by name; describe more complex techniques in the Methods section.*
☐	☒	A description of all covariates tested
☐	☒	A description of any assumptions or corrections, such as tests of normality and adjustment for multiple comparisons
☐	☒	A full description of the statistical parameters including central tendency (e.g. means) or other basic estimates (e.g. regression coefficient) AND variation (e.g. standard deviation) or associated estimates of uncertainty (e.g. confidence intervals)
☐	☒	For null hypothesis testing, the test statistic (e.g. *F*, *t*, *r*) with confidence intervals, effect sizes, degrees of freedom and *P* value noted<br>*Give P values as exact values whenever suitable.*
☒	☐	For Bayesian analysis, information on the choice of priors and Markov chain Monte Carlo settings
☒	☐	For hierarchical and complex designs, identification of the appropriate level for tests and full reporting of outcomes
☒	☐	Estimates of effect sizes (e.g. Cohen's *d*, Pearson's *r*), indicating how they were calculated

Our web collection on statistics for biologists contains articles on many of the points above.

Software and code

Policy information about availability of computer code

Data collection	No custom software was used
Data analysis	Excel (Office 365); OriginPro 2015, IVIS imaging systems, NIS-Elements (Nikon), Kaluza (Gallios, Beckman Coulter)

For manuscripts utilizing custom algorithms or software that are central to the research but not yet described in published literature, software must be made available to editors and reviewers. We strongly encourage code deposition in a community repository (e.g. GitHub). See the Nature Research guidelines for submitting code & software for further information.

Data

Policy information about availability of data

All manuscripts must include a data availability statement. This statement should provide the following information, where applicable:
- Accession codes, unique identifiers, or web links for publicly available datasets
- A list of figures that have associated raw data
- A description of any restrictions on data availability

Raw data supporting the findings of this study are available from the corresponding author on request.

Field-specific reporting

Life sciences study design

All studies must disclose on these points even when the disclosure is negative.

Sample size	The in vitro experiments described in this manuscripts were conducted at least 3 times. The sample sizes in animal studies were determined based on statistical analysis in the approved animal protocols.
Data exclusions	No data exclusion.
Replication	The experiments in this study were repeated and experimental findings were reproducible.
Randomization	In general, the animals were randomly assigned into different groups in two animal models (lung inflammation and bacterial infection).
Blinding	Investigators were not blinded.

Reporting for specific materials, systems and methods

We require information from authors about some types of materials, experimental systems and methods used in many studies. Here, indicate whether each material, system or method listed is relevant to your study. If you are not sure if a list item applies to your research, read the appropriate section before selecting a response.

Materials & experimental systems

n/a	Involved in the study	
☐	☒	Antibodies
☒	☐	Eukaryotic cell lines
☒	☐	Palaeontology and archaeology
☐	☒	Animals and other organisms
☐	☒	Human research participants
☒	☐	Clinical data
☒	☐	Dual use research of concern

Methods

n/a	Involved in the study	
☒	☐	ChIP-seq
☐	☒	Flow cytometry
☒	☐	MRI-based neuroimaging

Antibodies

Antibodies used	anti-Integrin b2 (C-4, Lot#A0814); anti-ICAM-1 (H-4, Lot# C2816); anti-GAPDH (G-9, Lot #C2514); anti-NUP153 (R3G1, Lot#B2516). All from Sant Cruz Biotechnology.
Validation	All antibodies were verified by the supplier and each lot has been quality tested.

Animals and other organisms

Policy information about studies involving animals; ARRIVE guidelines recommended for reporting animal research

Laboratory animals	Mice, CD-1, 5-8 weeks, male.
Wild animals	n/a
Field-collected samples	n/a
Ethics oversight	the Institutional Animal Care and Use Committee of Washington State University, USA.<br>the Institutional Review Board, WSU

Note that full information on the approval of the study protocol must also be provided in the manuscript.

Human research participants

Policy information about studies involving human research participants

Population characteristics	Adult healthy subjects
Recruitment	The participants were recruited by advertisement on the WSU campus
Ethics oversight	The Institutional Review Board, WSU

Note that full information on the approval of the study protocol must also be provided in the manuscript.

Flow Cytometry

Plots

Confirm that:

- [x] The axis labels state the marker and fluorochrome used (e.g. CD4-FITC).
- [x] The axis scales are clearly visible. Include numbers along axes only for bottom left plot of group (a 'group' is an analysis of identical markers).
- [x] All plots are contour plots with outliers or pseudocolor plots.
- [x] A numerical value for number of cells or percentage (with statistics) is provided.

Methodology

Sample preparation	For the apoptosis analysis, after treatments, neutrophils were suspended in the binding buffer and the reagent Alexa Fluor 488-Annexin V was added and incubated for 15 min. Then Propidium iodide was added, followed by flow cytometry analysis. For the neutrophil ratios in the lavages, the total cells were stained with anti-Ly-6G antibodies in PBS containing 0.1% (w/w) BSA and analyzed on the flow cytometer. For the bacterial phagocytosis assay, The mouse peritoneal macrophages were isolated and cultured overnight in RPMI1640 medium supplemented with 20% FBS. The adherent macrophages were digested and utilized for the assay. P. aeruginosa were opsonized with 10% mouse serum and stained with lipid dye DiO before the phagocytosis assay. 2×106 CFU bacteria were incubated with 105 macrophages with or without 33 ng/ml RvD1 and 10 µg/ml CAZ at 37oC for 60 min. The macrophages were then washed once with PBS and subject to analysis by flow cytometry.
Instrument	Gallios, Beckman Coulter
Software	Kaluza, version1.3
Cell population abundance	For the apoptosis assay, the abundance of the relevant cell populations is around 10-20% (in Figure 4J). The human neutrophils used in the experiments were analyzed with a purity over 95% by flow cytometry (Fig. S2). For the bacterial phagocytosis, the purity of macrophages was measured to be more than 90% by flow cytometry (Fig. 6G).
Gating strategy	Isotype antibodies were used for grating strategy.

- [x] Tick this box to confirm that a figure exemplifying the gating strategy is provided in the Supplementary Information.
